# The effect of parental age on the quantity and quality of offspring in *Syngnathus typhle*, a species with male pregnancy

**DOI:** 10.1111/eva.13755

**Published:** 2024-07-17

**Authors:** Freya Adele Pappert, Daniel Kolbe, Arseny Dubin, Olivia Roth

**Affiliations:** ^1^ Marine Evolutionary Biology Zoological Institute, Christian‐Albrechts‐Universität Kiel Kiel Germany; ^2^ Evolutionary Ecology of Marine Fishes Helmholtz‐Centre for Ocean Research Kiel (GEOMAR) Kiel Germany; ^3^ Institute of Clinical Molecular Biology (IKMB) Christian‐Albrechts‐Universität Kiel Kiel Germany

**Keywords:** ageing, gene expression, parental investment, pipefish, pregnancy, sex role

## Abstract

Parental age impacts offspring quantity and quality. Most prior research focused on maternal age. Since in most organisms the mother produces the costly eggs plus provides all or most parental care, it is difficult to distinguish maternal effects mediated via the egg from later maternal care. Here, we addressed the effects of parental age on offspring in *Syngnathus typhle*, a pipefish with male pregnancy. The divide between one parent producing the eggs and the second parent being the exclusive provider of parental care facilitates a distinction between the effects of parental age on egg quality versus parental age on early development. We fully reciprocally crossed young and old mothers and young and old fathers and assessed impact of parental age combination on offspring number, offspring size, and offspring gene expression patterns. Neither parental combination significantly influenced offspring size or male gestation duration; however, they influenced the number of offspring. Paternal, but not maternal, age strongly affected the offspring gene expression. Offspring from old fathers exhibited substantial changes in the expression of genes related to cell cycle regulation, protein synthesis, DNA repair, and neurogenesis. Our findings thus highlight the importance of gestation, as opposed to gamete production, in shaping the parental contribution to offspring development.

## 1 INTRODUCTION

The burden of parental care and the energy investment necessary for reproduction often differ between males and females. In many species, energy investment differs based on the size of the gametes (known as anisogamy), with eggs typically much larger than spermatozoa and therefore regarded as more expensive to produce (Janicke et al., 2016). This primary difference is believed to underlie sexual selection, with females bearing a higher cost in offspring investment compared to males, thereby becoming the choosy sex (Hayward & Gillooly, 2011). Furthermore, in numerous animal species, females also shoulder the responsibility of pregnancy and child‐rearing. Live‐bearing, or viviparity, represents one of the most costly forms of parental investment (Blackburn, 2015). This reproductive strategy enables the embryo to develop inside the mother's body until fully mature, providing protection from external threats and ensuring the best possible conditions for embryogenesis. Viviparity has evolved independently more than 150 times in vertebrates, including fishes, amphibians, reptiles, and once in mammals (Blackburn, 2015).

Across many taxa, parental age at the time of copulation affects the number of offspring produced, their fitness, and sometimes their ultimate lifespan (Lansing, 1947; Monaghan et al., 2020). With advancing age, there is an increased likelihood in many species that genetic mutations and epigenetic variations accumulate, telomeres are shorter, the immune system weakens, and altered cell communication induces cancer development, increasing the likelihood of having offspring with birth defects or decreased lifespan (Zhang et al., 2022). In the common shag (*Phalacrocorax aristotelis*), an avian species, chicks produced by older parents experienced greater telomere loss during their growth period (Heidinger et al., 2016). Older parents may inadvertently contribute to shorter offspring lifespans due to factors like increased frailty and accelerated ageing (Heidinger et al., 2016; Lansing, 1947). Studies in mice or Asian elephants revealed that the offspring from older mothers or even grandmothers had a shorter lifespan (Reichert et al., 2020; Tarín et al., 2005). The process of parental ageing and its associated physiological changes represents a constraint on parental influence, as it is largely beyond their control. In a hypothesized mechanism known as transgenerational plasticity, parents could potentially adapt their offspring's phenotype to the expected future environment (Bhandari, 2016; Donelson et al., 2018). This mechanism is believed to facilitate the acclimatization of embryos to their expected environment, potentially enhancing their overall fitness (Donelson et al., 2018). This rapid acclimatization primarily relies on epigenetic modifications, as opposed to genetic adaptations that require the accumulation of mutations over multiple generations (Duncan et al., 2014).

Experimental studies on parental investment and brood quality have focused primarily on maternal characteristics, seeing them as a major component of the offspring's own fitness, as females typically invest more not only in egg production but also in carrying and nurturing (Wolf & Wade, 2001). Nonetheless, the designation of sex (egg or sperm producer) and their respective sex role (i.e., parental care, courting, etc.) can be more ambiguous in the animal kingdom (Bachtrog et al., 2014). The realm of bony fish has seen the evolution of many forms of paternal care, from egg guarding, to mouth brooding, and varying degrees of pregnancy (Blumer, 1982; Smith & Wootton, 1995). Some of the most dedicated male parents can be accredited to the *Syngnathidae* family, which has evolved a range of male pregnant brooding forms from eggs carried ventrally on the males' tails (e.g., *Nerophis ophidion*) to full male pregnancies in brood pouches equipped with placenta‐like systems (i.e., *Hippocampus erectus*), providing the embryo with nutrients, oxygen, and immunological components (Beemelmanns & Roth, 2016; Ripley & Foran, 2009; Stölting & Wilson, 2007; Whittington & Friesen, 2020). Furthermore, this diverse family of colorful fish exhibits a remarkable range of reproductive behaviors, including various sex roles (from conventional to reversed), mating systems (monogamy, polygamy, and polyandry), and even prolonged gestational periods in some males (ranging from 9 to 69 days) (Rosenqvist & Berglund, 2011; Sloman & Buckley, 2011; Wilson et al., 2003). Despite the fascinating array of reproductive strategies in syngnathid, our understanding of how these diverse sex roles and associated life‐history trade‐offs influence offspring fitness remains limited. Gaining a deeper understanding of these dynamics holds significant implications for various fields, including evolutionary and reproductive biology, life‐history theory, and even conservation strategies aimed at managing populations with unique reproductive strategies.

Here, we used the broad‐nosed pipefish *Syngnathus typhle*, to address the effects of parental age on offspring fitness, mediated through gamete quality or gestation. This viviparous fish features male pregnancy with an inverted semi‐sealed brood pouch (Roth et al., 2012), and also exhibits sex‐role reversal, where females compete for access to males during the breeding season (Mobley et al., 2018). In this unique system, males are more reproductively constrained due to the size limitations of their brood pouches, while females produce more eggs on average than males can brood simultaneously (Braga Goncalves et al., 2010). The strength of sexual selection is reversed in this instance, with males being the choosier sex seeking out larger and more attractive females (Mobley et al., 2018). We aimed to investigate how maternal and paternal age affects offspring quantity and quality in this sex‐role‐reversed species with male pregnancy, ultimately permitting to disentangle who of the parents, that is, maternal or paternal cues, has a stronger influence on the offspring's phenotype. This unique model species allows us to disentangle the effects of egg and pregnancy, typically female‐specific traits, and explore the value of the eggs provided by the mother, as well as the role of the pregnant father in shaping offspring phenotypes.

This study aimed to address several key questions related to the impact of parental age and sex roles on offspring phenotype at birth in the unique reproductive system of *S. typhle*. Specifically, we investigated how increased paternal investment and paternal age influence offspring outcomes compared to maternal egg provisioning and maternal age. Specifically, we analyzed if the genetic history provided by the mother in the egg still outweighs the potential effects of the father's costly time and energy investment throughout pregnancy. Additionally, we sought to explore whether somatic age, often considered detrimental to reproduction and offspring health, holds true in a species where fertility increases with age. Fertility in *S. typhle* increases with age as individuals grow bigger, females will typically produce more eggs and males can harbor more embryos in their pouch (Berglund et al., 1986; Mobley et al., 2018). To address these questions, we conducted controlled mating experiments involving different combinations of parental age, including old males with old females (OM × OF), old males with young females (OM × YF), young males with old females (YM × OF), and young males with young females (YM × YF). We gathered quantitative data, such as the duration of pregnancy, the total offspring counts, and morphological measurements at birth. In parallel, we performed full transcriptome mRNA sequencing (RNA‐Seq) on at least five offspring from each mating pair to identify differentially expressed genes in the offspring based on the parental mating combination.

We hypothesized that the effects of paternal age may surpass those of maternal age in *S. typhle* due to the resource‐intensive nature of paternal investment, which is expected to vary based on the father's body size and life history. This research possesses two primary strengths. Firstly, the reproductive behavior of this species enables us to effectively distinguish the impact of parental age on offspring quality, whether mediated via egg or postfertilization care. Secondly, examining sex‐role reversal contributes to our overall comprehension of how sex roles affect various aspects of evolutionary processes.

## 2 METHODS

### 2.1 Sample collection

*Syngnathus typhle* were collected in April 2021 from two locations: the Limfjord in Lemvig, Denmark (56°33′47.239″ N; 8°17′49.805″ E) and the southwestern Baltic Sea in Falckenstein (56°33′47.239″ N; 8°17′49.805″ E). The pipefishes were brought to the GEOMAR Helmholtz Centre for Ocean Research, Kiel, where they were divided by sex and placed in 100 L tanks (50L × 40W × 51D) at either 30 ppt (Limfjord) or 18 ppt (Baltic) salinity, maintained at a temperature of 14°C. The fish received three anti‐parasite treatments to avoid importing detrimental parasites into our laboratory environments. This consisted of a combination of Malachitgrünoxalat (0.18 g/100 mL) and formaldehyde (2.06 g/100 mL), applied once on the first day and then once again on the third day. After which, to keep experimental conditions the same and to acclimate the pipefish collected from Lemvig to Baltic seawater, a slow filtered inlet drip was used over the course of a week till 18 ppt was reached for all pipefish. After which, all pipefish were kept for another week with the temperatures very slowly being increased to 18°C, simulating environmental temperature conditions optimal for mating.

Pipefish that were roughly 1 year old were categorized as young, and those above 2 years as old. The two age classes differ in the condition of their reproductive organs at the beginning of the reproductive season and in body size. In the early spring, when we collected the fish for this experiment, the cohort aged 2 years already displayed developed brood pouches, with females carrying eggs. In contrast, the first‐year cohort had not fully reached maturation, as evidenced by undeveloped pouches in males and slim females without eggs. However, during the incubation time (2 weeks), we could ensure that enough fish from the younger cohort did reach sexual maturation for mating. Before the start of the experiment, additional factors such as body mass and length (from snout to the end of the caudal fin) were measured to further assign age classes (Figure S1).

One nonpregnant male and one female from the same population were then placed together in 30 individual 100 L tanks (50L × 40W × 51D) based on their age, where they were kept for a 2‐week mating period. This time is more than sufficient to allow for the fish to mate, seeing as their typical reproductive window is quite brief. In total, we utilized 30 males and 30 females across both populations, comprising 14 old males (OM), 15 old females (OF), 16 young males (YM), and 15 young females (YF). There was a higher number of pairs from Lemvig with 23 pairs: five OM × OF, six OM × YF, six YM × OF, and six YM × YF, compared to Falckenstein with seven pairs: two OM × OF, one OM × YF, two YM × OF, and two YM × YF. The difference in the number of pipefish from each site was due to fewer fish being available at Falckenstein. Subsequently, the successful pregnant males were kept isolated in their respective tanks and the duration of each pregnancy was recorded. Once the offspring were born, five individuals from each parent were sampled and killed with an overdose of Tricaine mesylate (MS‐222; 500 mg/L; Sigma‐Aldrich), measured for total body length and weight. The whole body was immediately preserved in RNA later, as their organs were too small and translucent for individual dissection. The samples were stored at 4°C for 3 days before being transferred to −20°C for long‐term storage.

### 2.2 RNA isolation and mRNA sequencing

We used RNeasy Mini Kit (Qiagen–Venlo, Netherlands) to extract RNA from whole body tissue from 98 samples roughly five offspring from five different parents, resulting in the following amounts for each parental age combination: 25 OM × OF, 23 OM × YF, 25 YM × OF, and 25 YM × YF. We quantified the extracted RNA with a Peqlab NanoDrop ND‐1000 spectral photometer (Erlangen, Germany) and stored the samples at −80°C. BGI Tech Solutions (Hong Kong) performed library prep using the DNBSEQ Eukaryotic Strand‐specific mRNA library, followed by stranded mRNA sequencing on a DNBseq platform (150 bp reads, 25 M clean paired‐end reads per sample).

### 2.3 Statistical analysis

Statistical analysis was done in Rstudio v.4.2.2 (R Core Team, 2022). We initially assessed normal distribution of our body size, pregnancy duration, and offspring quantity data, then conducted separate two‐way ANOVAs for all three variables plus the length and weight of the adult pipefish that successfully mated and gave birth to characterize the sizes of our “young” and “old” parents (11 OMs, 14 OFs, 13 YMs, and 10 YFs). Any significant interaction we followed up with a Tukey's Honest Significant Differences (HSD) test. The HSD test allowed us to conduct a closer pairwise comparison between the different mating parental groups (i.e., OM × OF, OM × YF, YM × OF, and YM × YF).

With regard to the RNA‐Seq data, we performed a PERMANOVA (Permutational Multivariate Analysis of Variance Using Distance Matrices) using the “adonis2” function, “bray” method and family as strata. We did this analysis on logarithmically transformed count data to determine whether there were significant differences in gene expression between parental mating combinations and their sampling location. After normalizing for differences in library size, filtering out low counts, and scaling to counts per million (cpm) as described in point 2.4, we performed a regularized log transformation procedure (rlog) on the data for a principal component analysis (PCA) to detect group clustering and possible outliers (Figure S3). Two outliers (Figure S3) identified during PCA analysis were distorting the data distribution, affecting our analysis. We removed them to ensure robust data normalization and improve differential gene expression analysis accuracy.

### 2.4 Gene expression analysis

Quality control of the resulting reads from the Illumina sequencing was carried out using FastQC v.0.11.9 (Andrews, n.d.), and trimming was performed using Fastp v.0.20.1 (Chen et al., 2018). Subsequently, the reads were aligned to a whole genome assembly of *S. typhle* (BioProject ID: PRJNA947442) using STAR v.2.7.9a (Dobin et al., 2013). Transcript abundance was calculated using TPMCalculator (Alvarez et al., 2019). We used Orthofinder v.2.4.0 (Emms & Kelly, 2019) to assign functional information to annotated genes with a species list including the following accessions: *Takifugu rubripes* (rubripes.fTakRub1.2), *Syngnathus rostellatus* (GCF_901709675.1, fSynAcu1.2), *Syngnathus acus* (GCF_901709675.1, GCF_901709675.1_fSynAcu1.2), *Nerophis ophidion* (BioProject ID: PRJNA947442), *Oryzias latipes* (ASM223467v1), *Hippocampus erectus* (PRJNA613176), *Hippocampus comes* (H_comes_QL1_v1, GCA_001891065.1), *Lepisosteus oculatus* (LepOcu1), *Gasterosteus aculeatus* (BROADS1), and *Danio rerio* (GRCz11). Differential gene expression (DGE) analysis was performed on the RNA‐Seq data obtained from Illumina in Rstudio v.4.2.2 (R Core Team, 2022), using *S. acus* orthology annotations. The edgeR package v.3.40.2 (Robinson et al., 2010) was used for scaling to counts per million (cpm) and normalizing for differences in library size, genes with less than 10 counts in at least five libraries were discarded. We then normalized for composition bias by trimmed mean of M values (TMM) with *calcNormFactors* function, which calculates a set of normalization factors for each sample thereby eliminating composition biases between libraries. After this, we used the *limma* package v.3.54.1 (Ritchie et al., 2015), as a linear model‐based method that uses empirical Bayes approach and *voom* method (converts count data to continuous scale) to identify differentially expressed genes (DEGs). *Limma* is a software package useful in accounting for batch effects and mitigating other sources of technical variation, while *voom* has the best power of methods that control the type I error rate and is specifically designed to handle library sizes that vary by an order of magnitude or more (Law et al., 2014). This capability was crucial in this experiment because we wanted to reduce any batch effect and type I errors in DGE results due to pooling five individuals per parent and thus not being able to entirely avoid direct parental effects. The differential expression analysis comparing several groups was done by creating a matrix of independent contrasts; this way it was possible to perform a one‐way analysis of deviance (ANODEV) for each gene. Thereafter, we estimated coefficients of interest between groups to have log‐fold changes (logFCs) with the function *contrasts*. *fit*, and performed an empirical Bayes moderation of the estimated variance which shrinks sample variance and applies a moderate *t*‐test to identify DEGs. For further downstream analysis, we only used the resulting adjusted *p* values (Benjamin–Hochberg false discovery rate) below 0.05 to ensure that the observed treatment effects were real.

### 2.5 Orthology and enrichment analysis

A homology‐based search of *Danio rerio* was used with OrthoFinder to annotate DEGs for gene ontology analysis. The *Danio rerio* orthologues of the DEGs per contrast group were then uploaded to g:Profiler (last accessed in March 2023) to conduct functional profiling based on the offspring's parental mating age combination (OM × OF vs. YM × YF, OM × OF vs. YM × OF, OM × OF vs. OM × YF, YM × YF vs. YM × OF, YM × YF vs. OM × YF). We set the statistical domain scope to “Only annotated genes” and the significance threshold to “g:SCS threshold” at 0.05. We also treated numeric IDs as “ENTREZGENE_ACC” and limited the data sources to GO biological processes and biological pathways from the Kyoto Encyclopedia of Genes and Genomes (KEGG) and Reactome (REAC) databases. The results of the analysis are presented in Table S1.

## 3 RESULTS

### 3.1 Quantitative differences between offspring of different parental age combinations

Morphological measurements of the parents showed a difference in total body size between young and old females (adj.*p*.val <0.0001) and for weight (adj.*p*.val <0.0001), as well as young and old males (length in centimeters, adj.*p*.val <0.0001; weight in grams, adj.*p*.val <0.01). OFs had a mean size of 19.36 cm (SE = 0.19), YFs 14.53 cm (SE = 0.32), OMs 17.10 cm (SE = 0.42), and YMs 14.38 cm (SE = 0.30). These morphological distinctions provide a clear basis for categorizing the age classes. Figure S1A–D provides additional frequency histograms illustrating the differences of body weight and length of male and female parents for age.

**FIGURE 1:**
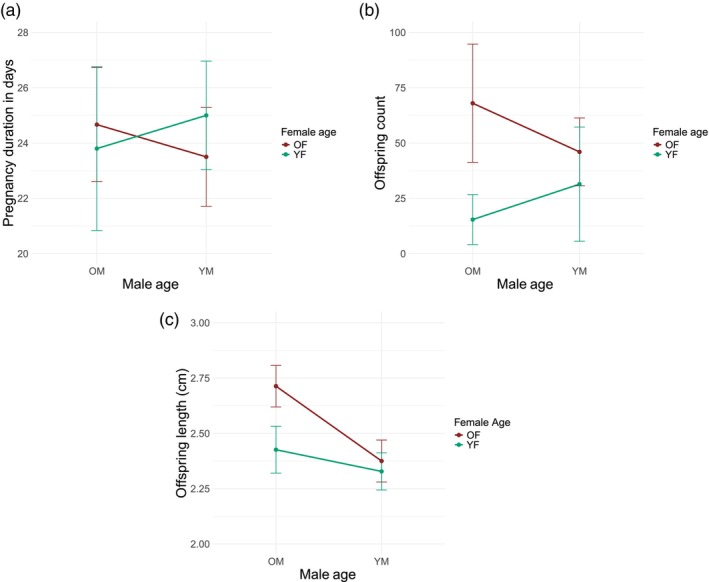
Interaction profiles of various quantitative measurements between different combinations of parental age of mating. Young male (YM) and old male (OM) are shown on the x‐axis, while the y‐axis represents the dependent variables. The turquoise line depicts the interactions with young female (YF), such as OM × YF and YM × YF. The second line in brown reflects the old females (OF), so combinations are OM × OF and YM × OF. The variation is displayed using error bars that represent the 95% confidence interval of the mean, calculated based on the standard error. (a) shows the duration of the pregnancy in days. (b) shows the total offspring count after birth. (c) depicts the total body length immediately after birth of five offspring per parent in centimeters. (See Figure S4 for separate boxplots for each individual parental combination).

Of the original 30 *S. typhle* couples, 24 successfully mated, males accepted and fertilized the eggs and were followed throughout pregnancy. We removed the six couples who did not successfully mate from any further analysis, these included one OM × OF, two YM × YF, one OM × YF (Lemvig population) and one YM × YF, one OM × YF (Falckenstein population). Of the 24 pregnant males, we tracked their gestational duration and found no significant difference between the four different groups of mating combinations (Figure 1a). The average pregnancy time for each group was as follows: OM × OF 24.7 days (SE = 0.80), OM × YF 23.8 days (SE = 1.07), YM × OF 23.5 days (SE = 0.76), and YM × YF 25 days (SE = 0.71). Offspring numbers differed between OM × OF and OM × YF (adj.*p*.val = 0.0013, Figure 1b) and between YM × YF and OM × OF (adj.*p*.val = 0.027). Overall, we found that the age of the mother significantly affected the offspring count (two‐way ANOVA, *p* < 0.001), whereas the age of the father had no significant effect (two‐way ANOVA, *p* = 0.6). There was, however, a significant interaction between maternal and paternal age on offspring count (two‐way ANOVA *p*.val = 0.03). OM × OF had the highest number of offspring with an average of 68 (SE = 10.4), and OM × YF had the lowest with an average of 15.4 (SE = 4.07), while the other two groups were somewhere in between with YM × OF 46 (SE = 6.49) and YM × YF 31.4 (SE = 9.30). From the resulting offspring that we collected (five per family) right at birth, we found no significant difference in weight, but a significant difference in body length for both the age of the father (two‐way ANOVA, *p*.val <0.0001), the mother (two‐way ANOVA, *p*.val = 0.003), and the interaction (two‐way ANOVA, *p*.val = 0.018). Post hoc analyses revealed significant differences in offspring size only between offspring from older parents and all other groups (OM × OF: OM × YF, adj.*p*.val = 0,0013; OM × OF: YM × OF, adj.*p*.val <0.0001; OM × OF: YM × YF, adj.*p*.val >0.0001; Figure 1c). Specifically, the mean body lengths of the offspring were 2.71 (SE = 0.046) cm for OM × OF, 2.43 (SE = 0.051) cm for OM × YF, 2.38 (SE = 0.047) cm for YM × OF, and 2.33 (SE = 0.041) cm for YM × YF.

### 3.2 Age of the male parent plays a critical role in offspring DGE

**FIGURE 2:**
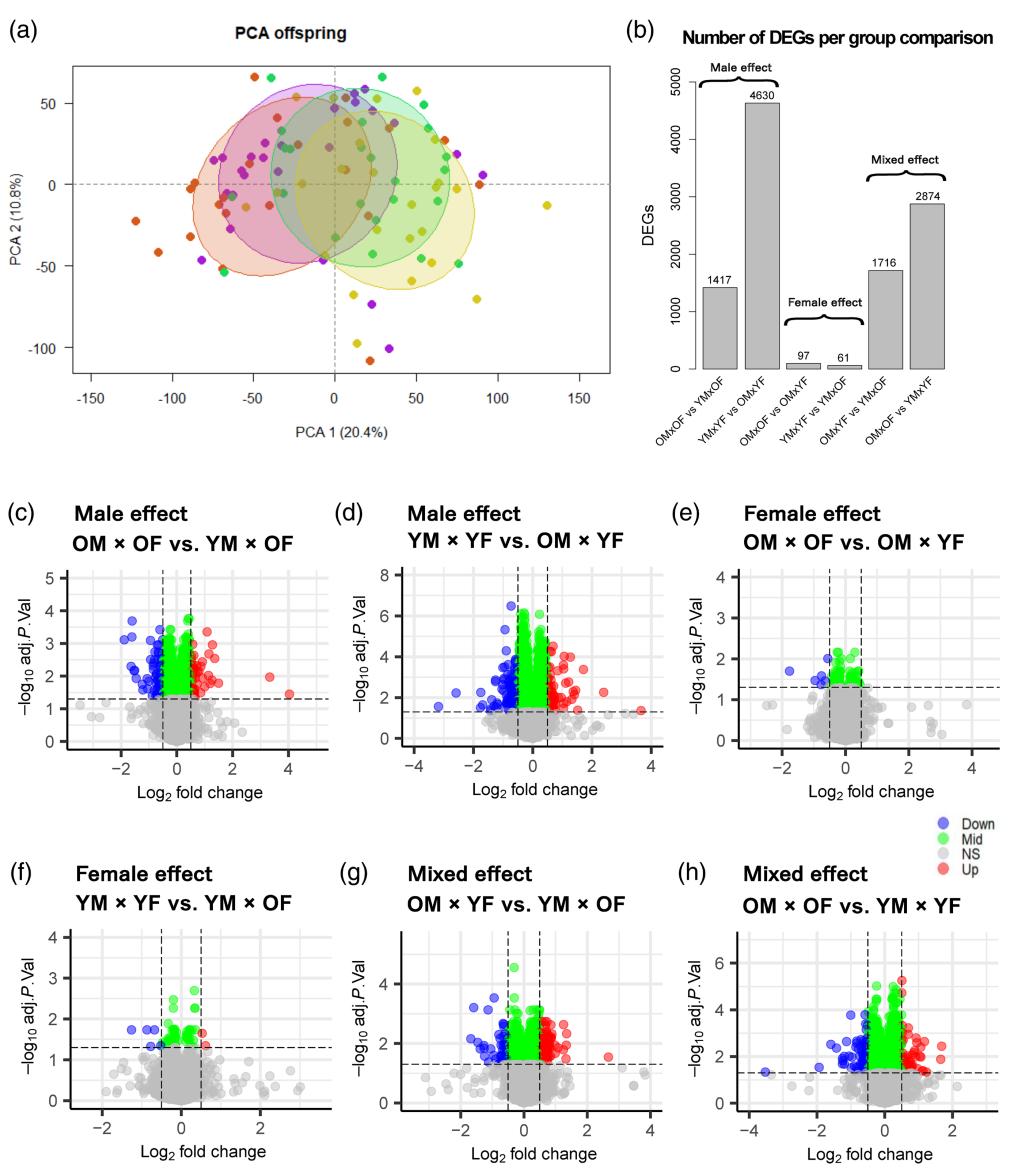
Results of the DGEs analysis. (a) PCA of pipefish offspring grouped by parental age combination: old male × old female (purple), old male × young female (brown), young male × old female (green), and young male × young female (yellow). (b) Bar graph showing the number of DEGs for each contrast comparison. (c–h) Volcano plots showing the DEG results for different contrast groups. The x‐axis represents the log2 fold change (logFC), and the y‐axis represents the negative log10 of the adjusted *p*‐value. Gray dots represent nonsignificant values (adj.*p*.val >0.05), while red dots indicate significant upregulated genes larger than logFC 0.05 in the first comparison listed, and blue dots indicate significant downregulated genes with a logFC below −0.5 also in the first comparison listed. For instance, in OM × OF versus YM × OF (Figure 2c), red dots indicate upregulated genes for OM × OF, and blue dots indicate downregulated genes in OM × OF compared to YM × OF. Green dots represent genes with a small logFC (between −0.05 and 0.05).

A PERMANOVA, assessing the influence of parental age combinations and location on the 26,072 transcripts from the sequenced *S. typhle* offspring, revealed that parental age combinations significantly influenced offspring gene expression (*p* = 0.013), while the difference between sampling locations did not (*p* = 0.116), presumably as both parental populations were kept under the same conditions for several weeks before mating. With a PCA, we saw no differences between parental populations in numerous PCs (Figure S2A–C) and were therefore confident that parental sampling location had no meaningful effect on the offspring, such that we grouped them together. When we distinguished between the four different parental age groups, we noticed that PC1 accounted for 20% of the variation, with the individual offspring clustering predominantly according to the father's age (Figure 2a). One can observe that on the left side of the plot, individual offspring clustered based on parent's from OM × OF and OM × YF combinations, here old male being the common denominator, while on the right, clustered offspring from YM × YF and YM × OF (Figure 2a), young males being the common denominator.

The analysis produced distinct numbers of DEGs for offspring from different parental age combinations (Figure 2b). When the age of only the male parent changed, a substantial number of DEGs were identified: 4630 genes for YM × YF versus OM × YF, and 1417 genes for OM × OF versus YM × OF (Figure 2b). In contrast, when the age of only the female parent changed, a much smaller number of DEGs were observed: 61 genes for YM × YF versus YM × OF, and 97 genes for OM × OF versus OM × YF (Figure 2b). This striking contrast underscores the smaller influence of female parental age on gene expression compared to male parental age in *S. typhle*. Figure 2a also visually illustrates this trend. When both parental ages changed, 2874 DEGs were identified for OM × OF versus YM × YF, and 1716 DEGs for OM × YF versus YM × OF (Figure 2b). Although this latter contrast is confounded by changes in both male and female ages, the overwhelming difference in DEGs associated with male age compared to female age suggests that the male contribution likely dominates. Additionally, the gene regulation direction for the various comparison groups is illustrated through log‐fold change (logFC) in volcano plots from Figure 2c–h. It is worth noting that with a change in female age, only very few genes exceeded the logFC ±0.5 threshold (Figure 2e,f), whereas with a change in paternal age, many more genes exhibited a higher logFC (Figure 2c,d), suggesting a more pronounced divergence in offspring from fathers of different ages.

The results of the functional profiling of the DEGs in each comparison group showed no enriched or overrepresented categories for the OM × OF versus OM × YF and YM × YF versus YM × OF groups due to the low number of DEGs. On the contrary, when the age of the father changed, we found pathways enriched in categories such as cell cycle, cell stress and response, protein synthesis and regulation, signal transduction, metabolism, and more (Tables 1 and S2). The majority of overlapping pathways were found between OM × OF versus YM × YF and YM × YF versus OM × YF (Table 1). In the first group (OM × OF vs. YM × YF), it was complicated to disentangle paternal from maternal effects, while in the second group (YM × YF vs. OM × YF), effects observed are driven by male age. One of the few pathways that we found to be enriched in both OM × OF versus YM × OF and YM × YF versus OM × YF was the extension of telomeres, which activates telomere enzymes to add new telomere repeats onto the ends of chromosomes (Blackburn, 2005). Upon examining the genes involved in the extension of telomere pathway, we found that out of a total of 26 genes belonging to this pathway, eight DEGs were present in OM × OF versus YM × OF and were all downregulated in offspring from YM × OF, while for the other group, there were 15 DEGs which were all upregulated for OM × YF (Tables S1 and S2). However, these genes were either *replication factors* or *polymerase subunits*, and we found that some overlapped with other pathways such as DNA replication and DNA repair, as frequently such genes are involved in a complex network of molecular processes contributing to the maintenance of genomic integrity.

**TABLE 1:** Summary table of most notable pathways from the gene set enrichment analysis.

OM × OF versus YM × YF	OM × OF versus YM × OF	YM × YF versus OM × YF	Pathway	Term ID	Description
Yes	Yes	Yes	DNA replication	REAC:R‐DRE‐69306	Cell cycle
Yes	Yes	Yes	Mitotic G1 phase and G1/S transition	REAC:R‐DRE‐453279	
Yes	Yes	Yes	S Phase	REAC:R‐DRE‐69242	
Yes	No	Yes	G1/S transition	REAC:R‐DRE‐69206	
Yes	No	Yes	Orc1 removal from chromatin	REAC:R‐DRE‐68949	
No	Yes	Yes	Extension of telomeres	REAC:R‐DRE‐180786	
Yes	No	Yes	Amide metabolic process	GO:0043603	Metabolism
Yes	No	Yes	Metabolism of RNA	REAC:R‐DRE‐8953854	
Yes	No	Yes	Macromolecule catabolic process	GO:0009057	
Yes	Yes	Yes	Cellular response to stress	GO:0033554	Cell stress response
Yes	No	Yes	p53‐dependent G1 DNA damage Response	REAC:R‐DRE‐69563	
No	No	Yes	Transcriptional regulation by TP53	REAC:R‐DRE‐3700989	
Yes	No	Yes	Translation	REAC:R‐DRE‐72766	Protein synthesis and regulation
Yes	No	Yes	Protein folding	GO:0006457	
Yes	No	Yes	Deubiquitination	REAC:R‐DRE‐5688426	
Yes	No	Yes	Regulation of RAS by GAPs	REAC:R‐DRE‐5658442	Cell signal transduction
Yes	No	Yes	Regulation of PTEN stability and activity	REAC:R‐DRE‐8948751	
Yes	No	Yes	Signaling by Hedgehog	REAC:R‐DRE‐5358351	
Yes	No	Yes	Signaling by WNT	REAC:R‐DRE‐195721	
Yes	No	Yes	FLT3 signaling	REAC:R‐DRE‐9607240	
Yes	No	Yes	MAPK6/MAPK4 signaling	REAC:R‐DRE‐5687128	
No	No	Yes	Neurogenesis	GO:0022008	Neuron development
No	No	Yes	Neuron differentiation	GO:0030182	
No	No	Yes	Neurotransmitter transport	GO:0006836	

*Note*: In the first three columns are the different parental group matches, where “yes” or “no” indicated if they had genes that were overrepresented in a particular pathway. Note that the comparison OM × YF versus YM × OF was excluded from Table 1 due to the confounding nature of parental combinations and the challenges in interpreting gene enrichment patterns in this context. For enriched pathways for all parental age combinations, please refer to Table S2.

There were enriched pathways pertaining to metabolism, protein synthesis, cell signaling and transduction that occurred only when the offspring had an old father (Tables 1 and S2). Overrepresented genes in pathways such as translation, protein folding, metabolism of RNA, amide metabolic process, regulation of *PTEN* (*Phosphatase and Tensin Homolog*) stability and of *RAS* (*Rat Sarcoma*) by GAPs (GTPase‐Activating Proteins), signaling by *Hedgehog* (*Hh*), and *MAPK* (*Mitogen*‐*activated protein kinase*) signaling were predominantly, if not exclusively, upregulated in offspring from older father's (Tables S1 and S2). Although there were some overlaps in the genes involved in these pathways, many of them encoded for mitochondrial ribosomal proteins, heat shock proteins, *cullin*, chaperones, prefolding subunits, proteasome activators or subunits, splicing factors, and *MAPK* (Tables S1 and S2).

Furthermore, the pathway for Orc1 removal from chromatin was enriched in OM × OF versus YM × YF and YM × YF versus OM × YF, with approximately 27 and 43 genes, respectively. Notably, 25 of the 27 Orc1‐related genes were upregulated in offspring from OM × OF, while all 43 genes were upregulated in OM × YF (Table S2). Orc1 is a crucial protein in the initiation of DNA replication in eukaryotic cells, and it has been found to have a strong impact on gene expression (Kara et al., 2015). On a similar note, offspring with an old male as father had enriched p53 pathways, which are involved in the response to DNA damage and other cellular stressors. The p53 protein is a transcription factor that regulates the expression of genes involved in cell cycle arrest, DNA repair, apoptosis, and senescence (Hafner et al., 2019). The majority of genes involved in this pathway were upregulated in offspring with an older father, regardless of the mother's age (Table S2). These included replication factors, cyclin‐dependent and checkpoint kinases, and tp53‐regulating kinases (Table S1). Furthermore, *lamtor3*‐*5* were upregulated, which are important components of the Ragulator‐Rag complex, which is responsible for regulating cell metabolism, trafficking in eukaryotic cells, and also the mTOR pathway, as it responds to amino acid levels in the cell (Zhang et al., 2017).

We found GO terms for neurogenesis, neuron differentiation, neurotransmitter transport, and so forth (Table 1 and S2), only for DEGs between YM × YF versus OM × YF. In particular, for neurogenesis alone (pathway includes 900 genes), there were 200 DEGs associated with this pathway (in YM × YF vs. OM × YF), of which 177 were upregulated in offspring from YM × YF (Tables S1 and S2).

## 4 DISCUSSION

Our study aimed to research how maternal and paternal age at reproduction affects offspring quantity and quality in *S. typhle*, a sex‐role reversed species with male pregnancy. We observed major differences in the number of offspring at birth based on parental age (Figure 1b), despite male and female age not affecting pregnancy time or duration (Figure 1a). Having both parents being older led to the highest number of offspring, which can be attributed to the larger brood pouch of male pipefish and the ability of larger females to produce more eggs. In contrast to our expectation, the lowest offspring number was produced by couples with an old father and a young mother, rather than by couples with two young parents (Figure 1b). The higher offspring count observed in YM × YF pairs compared to OM × YF pairs may be attributed to a combination of factors, including female egg production capacity and potential mate choice preferences. Research by Jones et al., (2000) found that male pipefish with access to larger females successfully mated with a mean of 1.3 females, whereas those with access to smaller females mated with a mean of 2.1. This indicates that male pipefish tend to mate with multiple younger females, potentially due to their lower egg production capacity. However, the influence of mate choice and mating preferences cannot be overlooked. *S. typhle*'s polygamous mating behavior means that males mate with multiple females, and embryos from different mothers can be found in a single pregnancy (Vincent et al., 1995). Male pipefish may exhibit selectivity in mate choice, preferring partners of similar age or size, or they may be less willing to accept eggs only from younger females, instead waiting to fill their brood pouches to capacity when encountering a larger and higher quality mate (Berglund et al., 1986). This selective behavior could contribute to the observed differences in offspring count between YM × YF and OM × YF pairs. We also observed a significant difference in body size measurements of the newly born juvenile pipefish between OM × OF and all other groups (Figure 1c). As egg size correlates positively with female body size in pipefish, having an older mother may likely provide a developmental advantage, as she will provide larger and more nutrient‐rich eggs, resulting in larger embryos (Braga Goncalves et al., 2011). In the wild, bigger is generally better, as the offspring will be less vulnerable to predators, can swim faster for resources, and are more competitive (Bashey, 2008; Dial et al., 2016).

Examination of transcriptome‐wide DGE revealed that the numerical disparity in DEGs among offspring from various combinations of parental age was primarily driven by the paternal lineage, with exceptionally many DEGs found when comparing offspring with both young parents (YM × YF) with those differing only in an older father (OM × YF) (Figure 2b). Interestingly, a PCA showed that the offspring grouped according to their father's age (Figure 2a), regardless of the age of the mother. These results suggest that paternal age and his experienced life history, influence more strongly gene expression in *S. typhle* offspring, compared to the information provided by the mother in the gametes. While the mother is in contact with the eggs during their development in her ovaries, the father's prolonged contact with the embryos during pregnancy exerts a greater influence on gene expression. Such prolonged contact during embryogenesis potentially allows for the transmission of nongenetic information, such as immune history and microbial communities, which could impact offspring phenotype and possibly influence their survival and reproductive success in the future (Bhandari, 2016; Burton & Metcalfe, 2014; Donelson et al., 2018; Roth et al., 2012; Tanger et al., 2016). Therefore, pipefish offspring may generally profit from the increased size and age of the mother by starting with a larger embryo, but their gene expression at birth and phenotype are likely more heavily influenced by the father.

Research in species with female pregnancy has shown that the mother has a stronger effect on offspring phenotype, as information passed from her ecological environment to the offspring is a more truthful signal (i.e., salinity, temperature, food availability, etc.) compared to the phenotype passed down from the father (Hagmayer et al., 2018; Wells, 2003). Yet, in such cases, one cannot differentiate whether the signal is due to the information provided in the egg or truly the increased parental care (i.e., pregnancy), as the two are intermingled within the female. Precisely in light of this, our experimental design allowed us to cleanly separate the effects of parental age on the egg versus postfertilization care, providing valuable insights into the mechanisms underlying offspring phenotype determination. Our results indicate that offspring DGE is driven by the paternal lineage, suggesting a significant influence of postfertilization care, such as pregnancy, on gene regulation. At this point in time, we cannot with full certainty state whether the parent‐specific gene expression is controlled by the father or the offspring. We acknowledge the complexity of these effects, as the mechanisms underlying such transgenerational effects could involve a combination of epigenetic control, vertical transfer of immunological components, or even environmental factors present in the microbial pouch environment. Furthermore, David Haig's work on parent–offspring conflict and genomic imprinting highlights the intricate interplay between maternal and paternal factors in shaping offspring development (Haig, 2014). Theories of parent–offspring conflict and genomic imprinting, posit that imprinted expression of genes reflects a conditional strategy, where alleles adopt matrigenic and patrigenic roles in different evolutionary contexts (Haig, 2014). Natural selection favors imprinted expression when conditional (imprinted) strategies outperform unconditional (unimprinted) ones. Moreover, the concept of parent‐specific gene expression suggests that differential expression is an adaptation of the imprinted gene itself, rather than the genes responsible for the imprinting process.

As the paternal age appears to count for more in affecting offspring gene expression, are older *S. typhle* fathers truly detrimental to offspring health? Functional profiling of DEGs revealed that the father's age was mostly influencing cell cycle, stress response, protein synthesis and regulation, signal transduction, and metabolism of the offspring (Table 1). However, we found that all 43 DEGs included in the Orc1 removal from chromatin pathway were upregulated in offspring with older male parents (Table S1 and S2), and as removal of Orc1 has been shown to cause defects in DNA replication and loss of integrity of chromatin structure (Kara et al., 2015), these changes can affect negatively gene expression and other cellular processes that depend on chromatin organization. Moreover, we also found many DEGs between YM × YF versus OM × YF, that were involved in various cellular processes, such as protein synthesis and regulation, signaling by Hh, MAPK6/MAPK4 signaling (aka ERK3/ERK4), regulation of *PTEN* stability, and regulation of *RAS* by GAPs, majority of which were upregulated offspring from OM × YF (Tables 1 and S2). These pathways are primarily involved in growth and developmental processes, suggesting that the age of the older father at copulation may negatively impact offspring cell division rates or potentially serve to mitigate DNA damage in response to external stressors, both of which could be linked back to telomerase activation (Blackburn, 2005; Robinson & Schiemann, 2022). Studies have shown that de novo mutations in germ cells that occur at higher rates in males, are then inherited and could subsequently lead to dysregulation of numerous cellular functions (Goldmann et al., 2019). Unfortunately, limited research has been conducted specifically on the influence of parental age on these pathways. However, their biological role is known and could be relevant in offspring development. Hh signaling is particularly crucial in embryonic development, organogenesis, and the renewal of various stem cell populations (Briscoe & Thérond, 2013). The commonly denominated atypical MAP kinase ERK3/ERK4 pathway is lined to having both pro‐ and anti‐oncogenic properties in various animal models (Kostenko et al., 2012). ERK/MAPK pathway is also known to play an important role in the regulation of neuronal survival and synaptic plasticity (Hutton et al., 2017). The latter fit together with the results from the enrichment of pathways involved in neurogenesis and neuron differentiation between YM × YF versus OM × YF (Table 1), in which 200 DEGs involved in the neurogenesis pathway alone had the vast majority (177 genes) upregulated in YM × YF (Tables S1 and S2).

Nevertheless, we found that out of the 35 DEGs involved in the PTEN pathways between YM × YF versus OM × YF, there were 33 upregulated in offspring with OM × YF (Table S2). *PTEN* encodes for a protein that plays a critical role in regulating cell growth and survival by inhibiting the PI3K/AKT signaling pathway (Keniry & Parsons, 2008), which in turn is crucial for regulating genetic stability and factors such as activating mTOR kinase inducing growth and translation, or by inhibiting transcription factors like FOXO family members thereby activating cell cycle progression and apoptosis (Keniry & Parsons, 2008). Activation of members of the mTOR and inhibition of FOXO family have a negative impact on health and longevity (Efeyan et al., 2015; Schaible & Sussman, 2013). Therefore, PI3K/AKT is involved in driving tumor progression and upregulation of *PTEN* leads to its suppression, and in this instance, offspring from young fathers are at a disadvantage compared to those from old fathers. Furthermore, we found that several downstream targets of TP53 were upregulated in offspring with young fathers compared to offspring from old fathers, in particular two *inducible tp53 proteins* and two *apoptosis*‐*stimulating p53 proteins* (Table S1). TP53 is a transcription factor regulating the expression of a large number of genes involved in various cellular processes, including apoptosis, DNA repair, and cell cycle arrest (Hafner et al., 2019). There are synergistic interactions between *PTEN* and *p53*, with the latter binding to *PTEN* promoter regions and regulating their expression (Keniry & Parsons, 2008). Also, the enriched pathway for regulation of *RAS* by GAPs, in DEGs from YM × YF versus OM × YF, can function both as either a tumour suppressor or as an oncogene depending on the genetic variants within the gene, seeing as *RAS* gene uncontrolled activation can lead to the development of tumors (Gimple & Wang, 2019) and GAP can downregulate said *RAS* genes (Bos et al., 2007). It would appear in this instance, that offspring from young males may have a propensity for developing tumors and have an upregulated cell cycle activity. Unfortunately, we cannot confirm this with biopsies thus limiting assumptions on possible negative health effects being passed on by either older or younger pregnant fathers. In the future, we aim to follow‐up on the morphological development of the offspring and possible health deterioration or tumorigenesis.

Overall, we find a complex interplay between paternal age and offspring health in *S. typhle*. While the upregulation of genes involved in Orc1 removal from chromatin in offspring from older fathers may indicate an increased risk of DNA replication defects and loss of chromatin integrity, it is important to consider potential compensatory mechanisms. For instance, the upregulation of PTEN‐related transcripts in these offspring could inhibit the PI3K/AKT pathway, potentially reducing the risk of tumorigenesis and conferring long‐term benefits. It is worth noting, however, that higher *PTEN* activity has also been implicated in influencing neuronal survival and growth, contributing to various neurodegenerative disorders (Ismail et al., 2012). Given the enrichment of neuronal developmental pathways among differentially expressed genes between offspring from younger and older fathers (e.g., YM × YF vs. OM × YF), with numerous genes downregulated in offspring having both old parents (Table S1), further research is warranted to elucidate the potential impacts of PTEN on embryonic neurogenesis. We propose that the importance of pregnancy in transgenerational plasticity in pipefish may be attributed to the distinct life histories experienced by older fathers, including potential exposure to pathogens or environmental stressors (i.e., nutrient availability, temperature fluctuations), modulating offspring gene expression. Nonetheless, we also find that offspring from younger fathers may experience detriments, albeit through different mechanisms. The complexity of our results highlights the challenges in making clear predictions regarding their consequences on offspring. Future research should go beyond birth and begin to assess the consequences of parental age for offspring performance. The crosses should be repeated under alternative environmental conditions to determine the broader implications of parental age on offspring performance.

This study delves into the less‐explored domain of parental age effects in sex‐role reversed fish species, contributing valuable insights into the intricate relationship between parental age, resource allocation, and transgenerational phenotypic plasticity. Our findings highlight the important role of pregnancy (i.e., parental care investment) and the parental life history, encompassing potential exposure to pathogens or environmental stressors, in influencing gene expression and the ultimate phenotype of the offspring. It is crucial for researchers to move beyond traditional animal models that oversimplify ageing as a binary division between male and female reproductive systems, and instead, we must acknowledge the complexity of nature, where gender roles can be fluid and ageing can be influenced by trade‐offs in life history. We also underscore the importance of incorporating life‐history information, such as parental age and reproductive experience, into conservation programs. By recognizing the nuanced interplay between life‐history traits and genetic outcomes, conservationists can develop more tailored and effective management plans to safeguard the long‐term survival and evolutionary potential of endangered or vulnerable species.

## FUNDING INFORMATION

Financial support was provided by the Deutsche Forschungsgemeinschaft (DFG, German Research Foundation) through the Research Training Group for Translational Evolutionary Research (RTG 2501 TransEvo).

## CONFLICT OF INTEREST STATEMENT

The authors declare no conflicts of interest.

## Supporting information


Appendix S1

Figure S1

Figure S2

Figure S3

Figure S4

Table S1

Table S2

## Data Availability

Supplemental information includes additional figures (Figures [Link], [Link], [Link], [Link]), the *Danio rerio* orthologues of the significant DEGs in offspring pipefish between the various contrast groups (Table S1) and the gene set enrichment analysis results (Table S2). Analysis scripts were adapted from the pipeline outlined in the 2018 RNA‐Seq Workshop (https://github.com/ucdavis‐bioinformatics‐training/2018‐June‐RNA‐Seq‐Workshop) and we incorporated methodologies described in the Bioconductor limma user manual (https://www.bioconductor.org/packages/devel/bioc/vignettes/limma/inst/doc/usersguide.pdf). The raw sequencing data and metadata used in this study are available from the National Centre for Biotechnology Information (NCBI) Sequence Read Archive (SRA) under BioProject ID PRJNA973859 and accession number SUB13263866.
