# Supplementary material for: The effect of parental age on the quantity and quality of offspring in Syngnathus typhle, a species with male pregnancy
Source: Evol Appl. 2024 Jul 17;17(7):e13755. doi: 10.1111/eva.13755 (PMC11254578; doi:10.1111/eva.13755)

The effect of parental age on the quantity and quality of offspring in *Syngnathus typhle*, a species with male pregnancy.

Supplementary material

**Fig. S1**: Represented are density histograms of morphological measurement results of length and weight of pipefish parents. The age groups are divided into old male (OM) in dark blue, young male (YM) in light blue, old female (OF) in dark pink, and young female (YF) in pink. The x-axis represents the measured parameters of either weight in grams (g) or length in centimeters (cm). Figure A) shows the length of the male pipefish, the mean total body size for OM was 17.1 cm (SE = 0.42) and for YM 14.38 cm (SE = 0.30), (OM vs. YM [cm], TukeyHSD, p.adj.p < 0.0001). B) Density histogram for male weight with OM having a mean body weight of 1.96 g (SE = 0.16) and YM of 1.27 g (SE = 0.10), (OM vs. YM [g], TukeyHSD, p.adj < 0.01). Plot C) shows female total body length with OF average being 19.36 cm (SE = 0.19), and for YF 14.53 cm (SE = 0.32), (OF vs. YF [cm], TukeyHSD, p.adj < 0.0001). D) shows female body weight with OF average weight of 3.28 g (SE = 0.11), and YF of 1.41 g (SE = 0.14), (OF vs. YF [g], TukeyHSD, p.adj < 0.0001).

A B


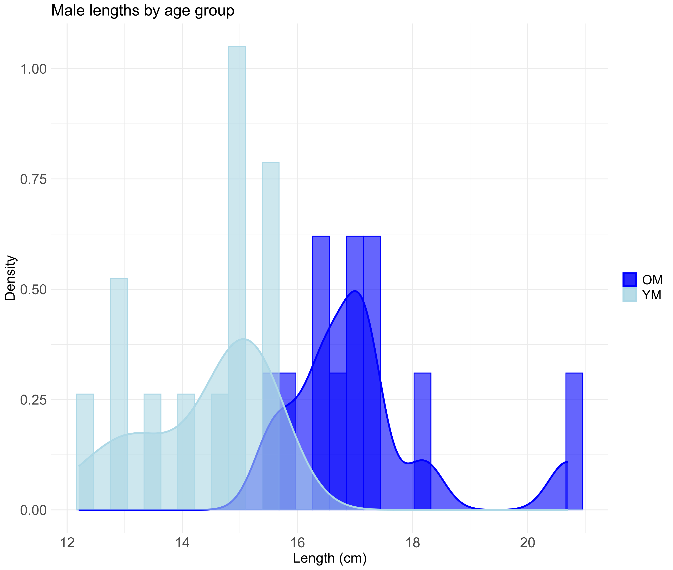

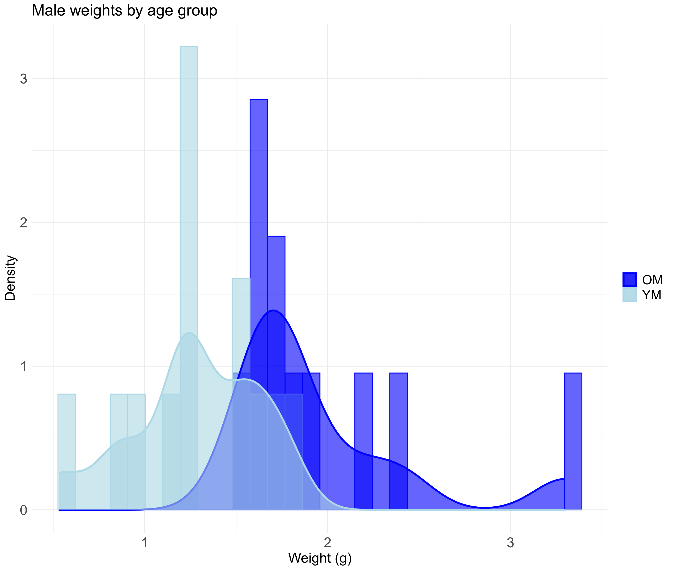


C D


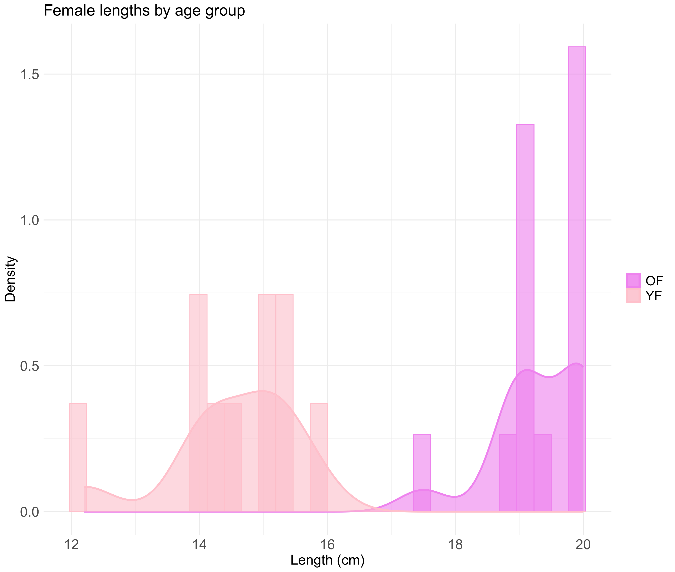

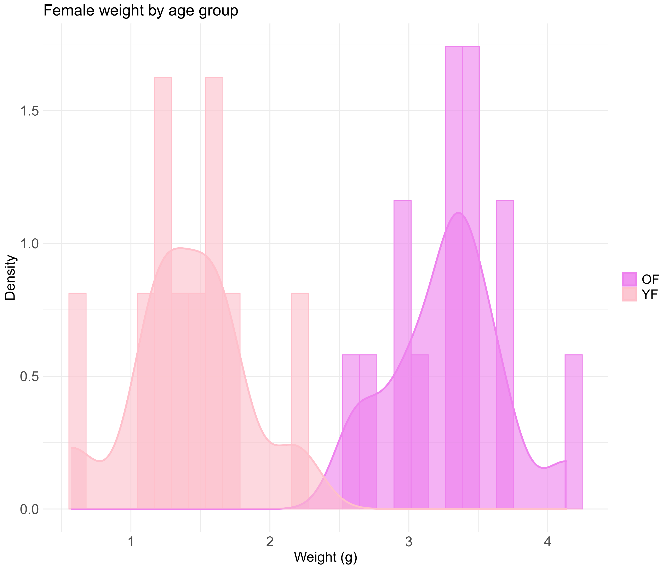


**Fig. S2 (A-C)**: PCA plots showing PC1 to PC4 on rlog transformed count data of all the offspring individuals based on if their parents came from Falckenstein (red) or Lemvig (blue). No obvious clustering was detected between offspring from different parental locations, which corroborated our PERMANOVA analysis (P > 0.05) that parental population didn’t have an effect.


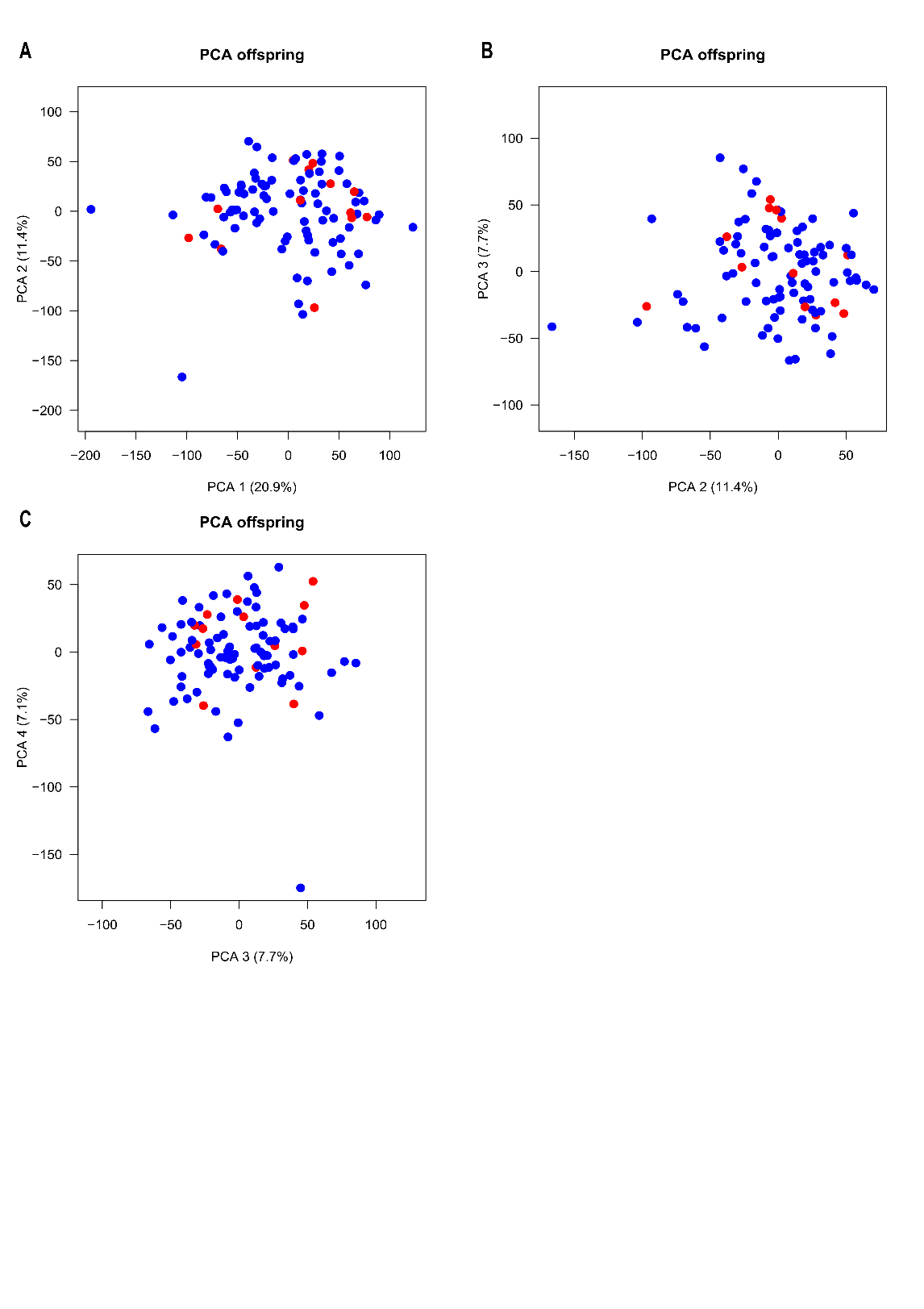


**Fig. S3**: The figure shows a principal component analysis (PCA) of rld transformed counts of pipefish offspring, grouped by parental combination: OMxOF (purple), OMxYF (brown), YMxOF (green), and YMxYF (yellow). PCA1 in all likelihood reflects the difference between offspring from old and young fathers. However, we detected two distinct outliers, one from OMxYF and one from YMxOF. These samples were distorting the distribution of the data and we decided to remove them for differential gene expression analysis to facilitate data normalization, enabling better analysis and modeling.


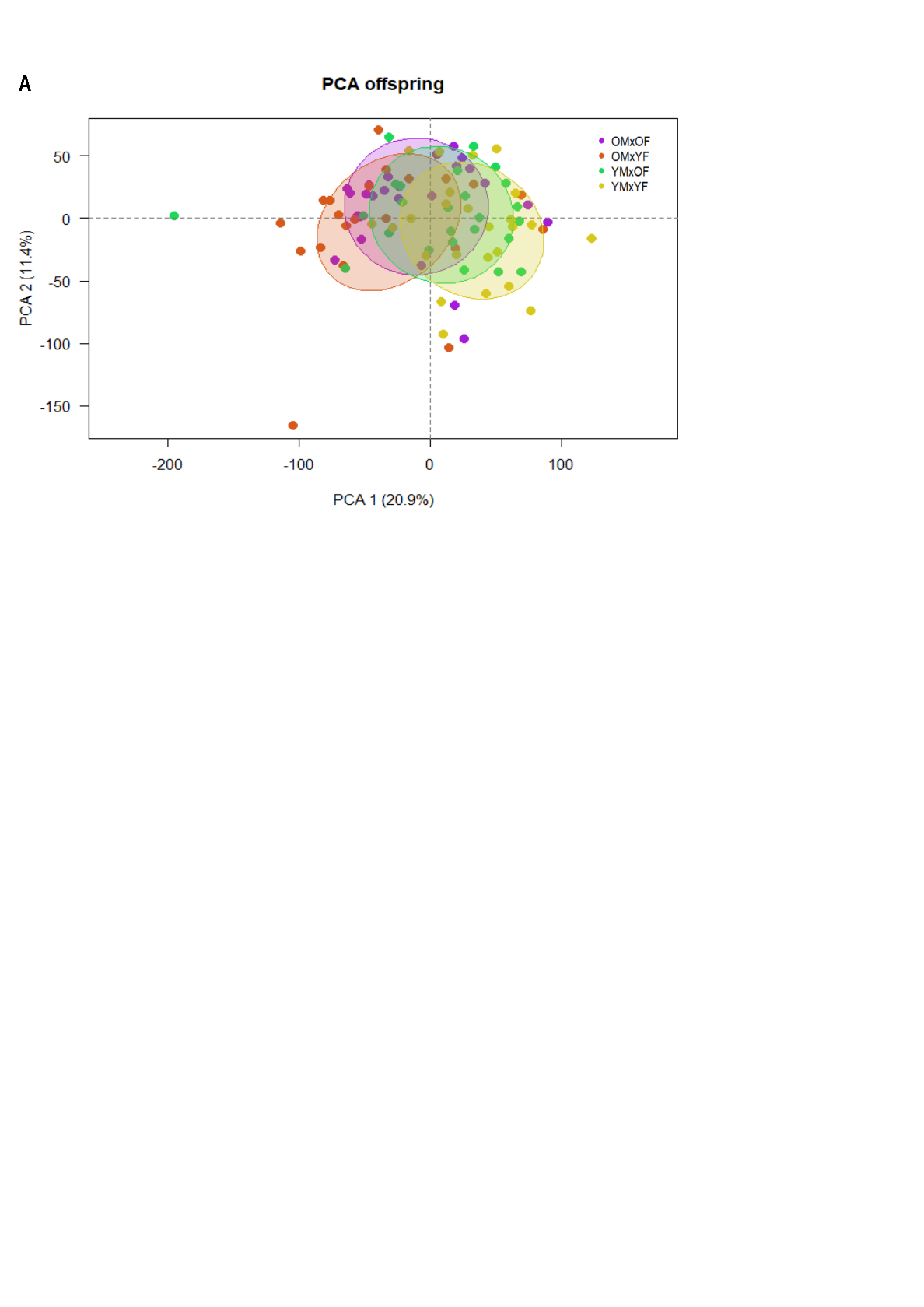


**Fig. S4:** Boxplots of various quantitative measurements between different combinations of parental age of mating. In purple old male with old female, in orange old male with young female, in green young male with old female, and yellow young male with young female. Highly significant P values from HSD test are marked above the boxplots with a line designating the comparison groups. A) Boxplot of duration of the pregnancy in days. B) Boxplot of the offspring count per parental group. C) Boxplot of total body length immediately after birth of five offspring per parent in centimetres.


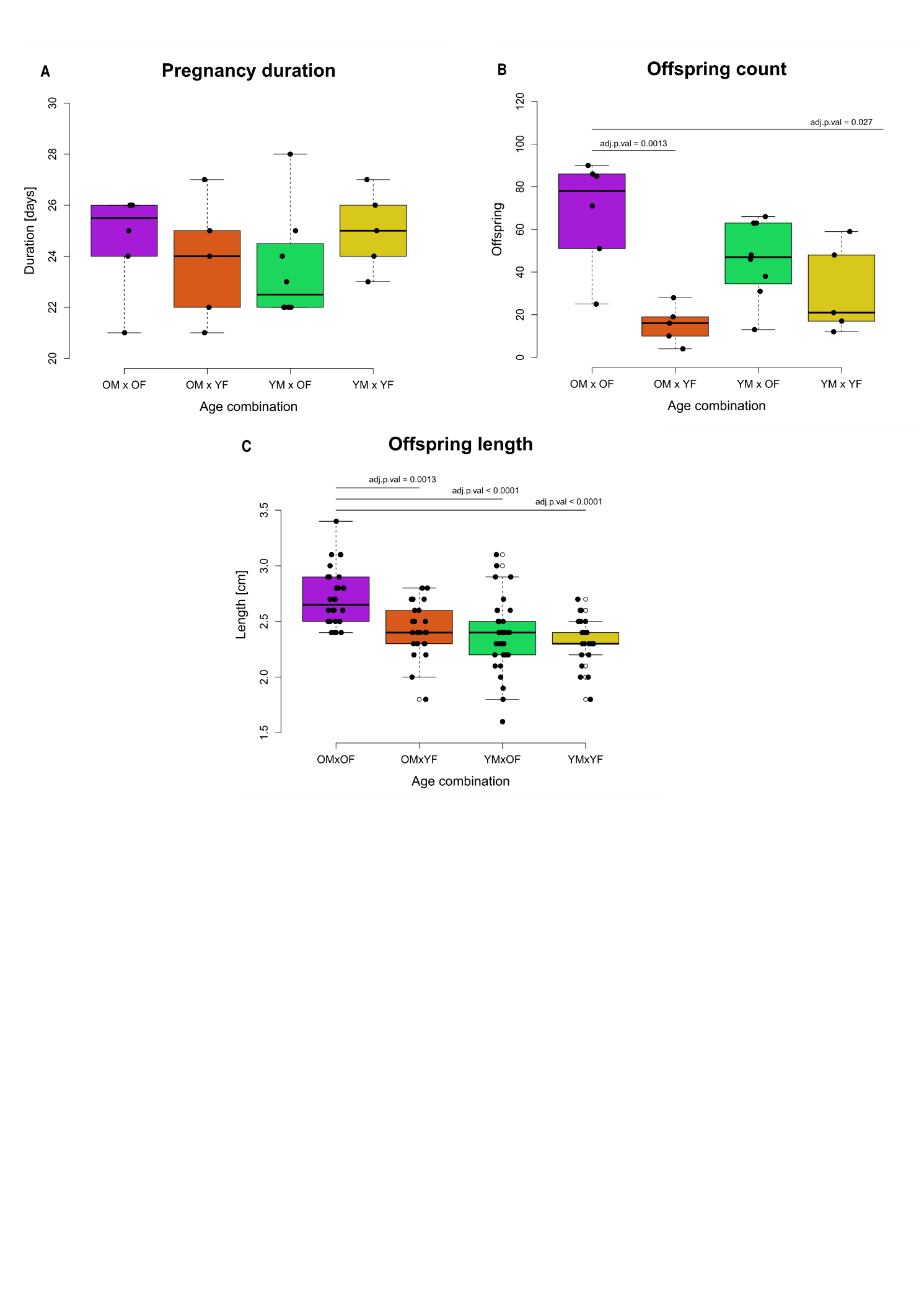

Supplement: Supplementary file 1 — Appendix S1 [file EVA-17-e13755-s001.docx]
