# Supplementary figures and images for: The effect of parental age on the quantity and quality of offspring in Syngnathus typhle, a species with male pregnancy
Source: Evol Appl. 2024 Jul 17;17(7):e13755. doi: 10.1111/eva.13755 (PMC11254578; doi:10.1111/eva.13755)

Male lengths by age group

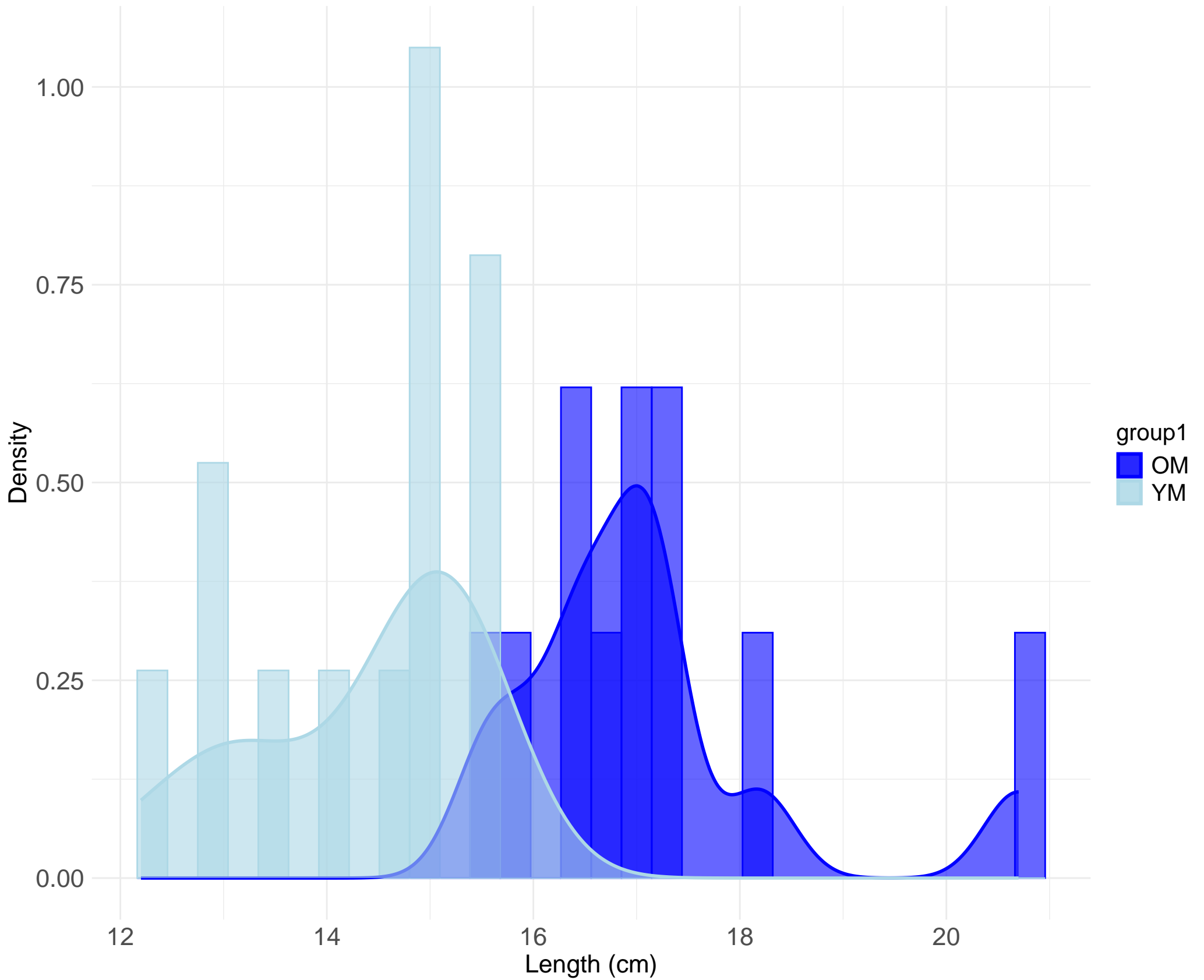

Supplement: Supplementary file 2 — Figure S1 [file EVA-17-e13755-s005.zip › Figure_S1A.pdf]

Male weights by age group

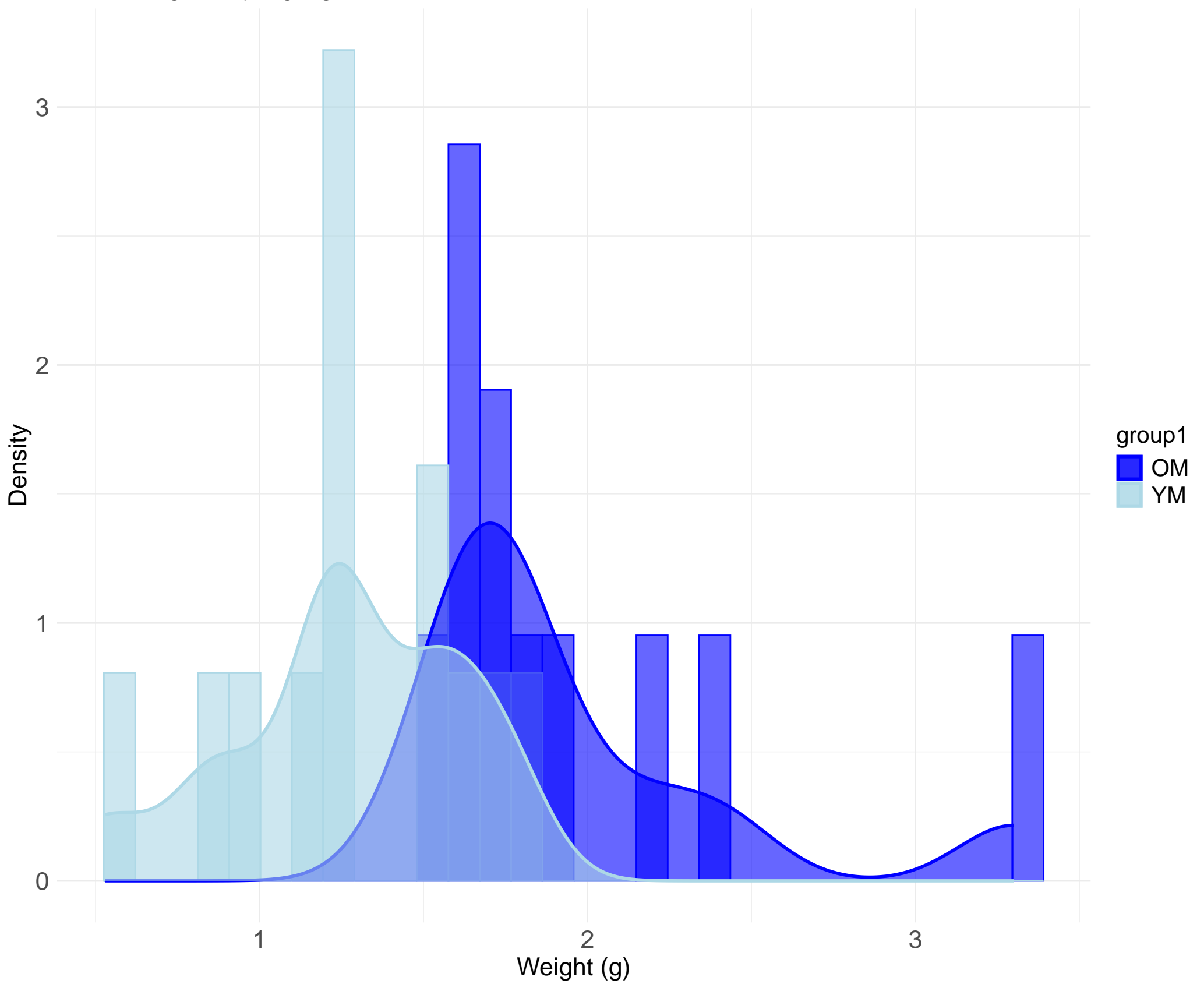

Supplement: Supplementary file 2 — Figure S1 [file EVA-17-e13755-s005.zip › Figure_S1B.pdf]

Female lengths by age group

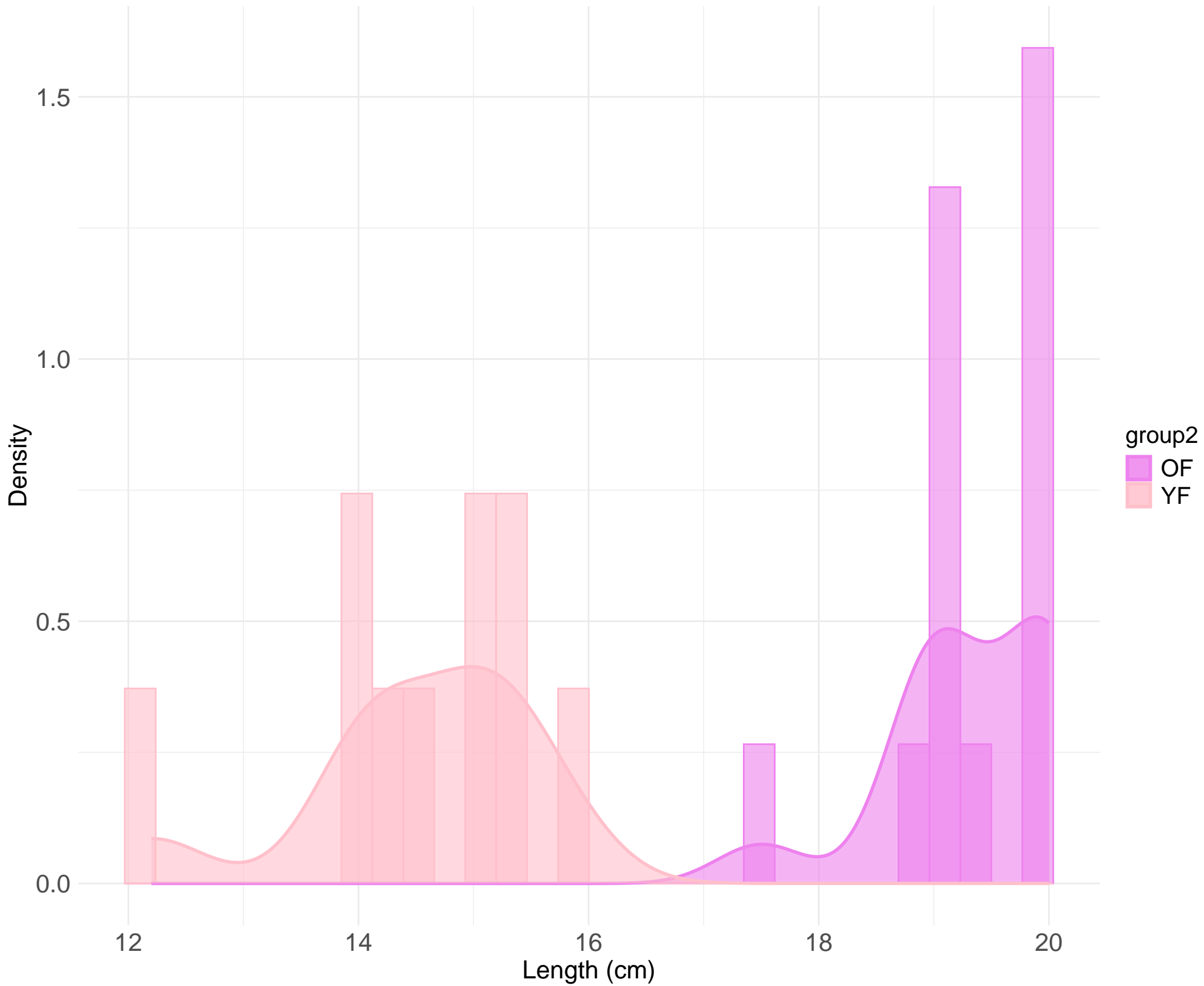

Supplement: Supplementary file 2 — Figure S1 [file EVA-17-e13755-s005.zip › Figure_S1C.pdf]

Female weight by age group

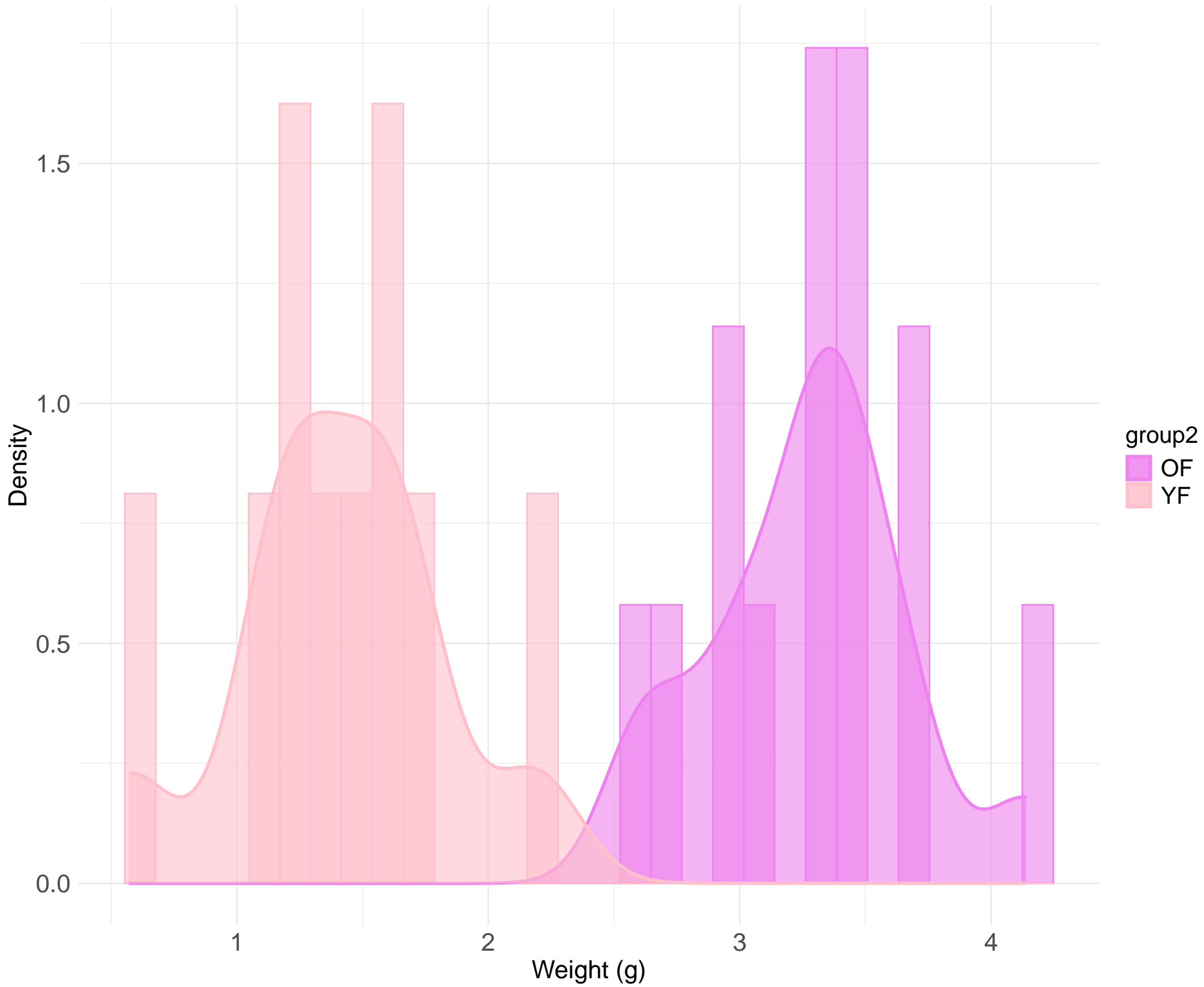

Supplement: Supplementary file 2 — Figure S1 [file EVA-17-e13755-s005.zip › Figure_S1D.pdf]

# PCA offspring

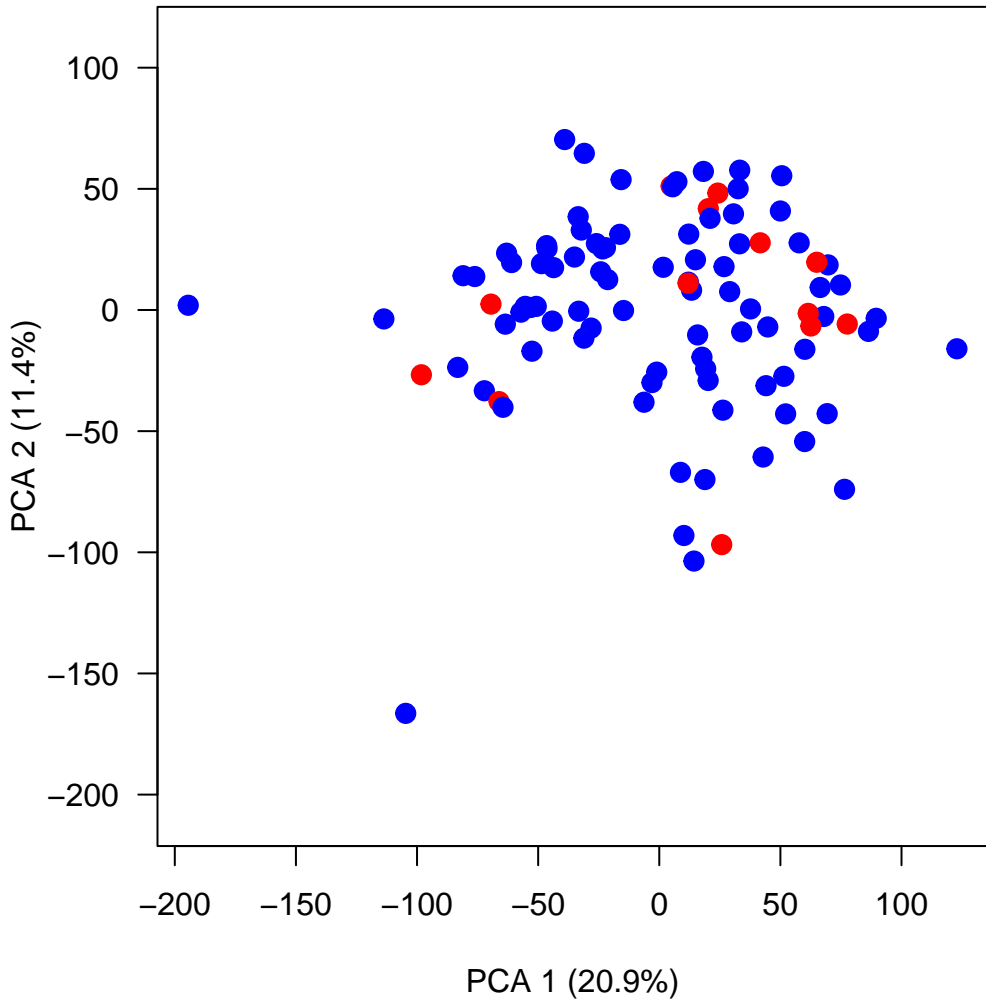

Supplement: Supplementary file 3 — Figure S2 [file EVA-17-e13755-s004.zip › Figure_S2A.pdf]

# PCA offspring

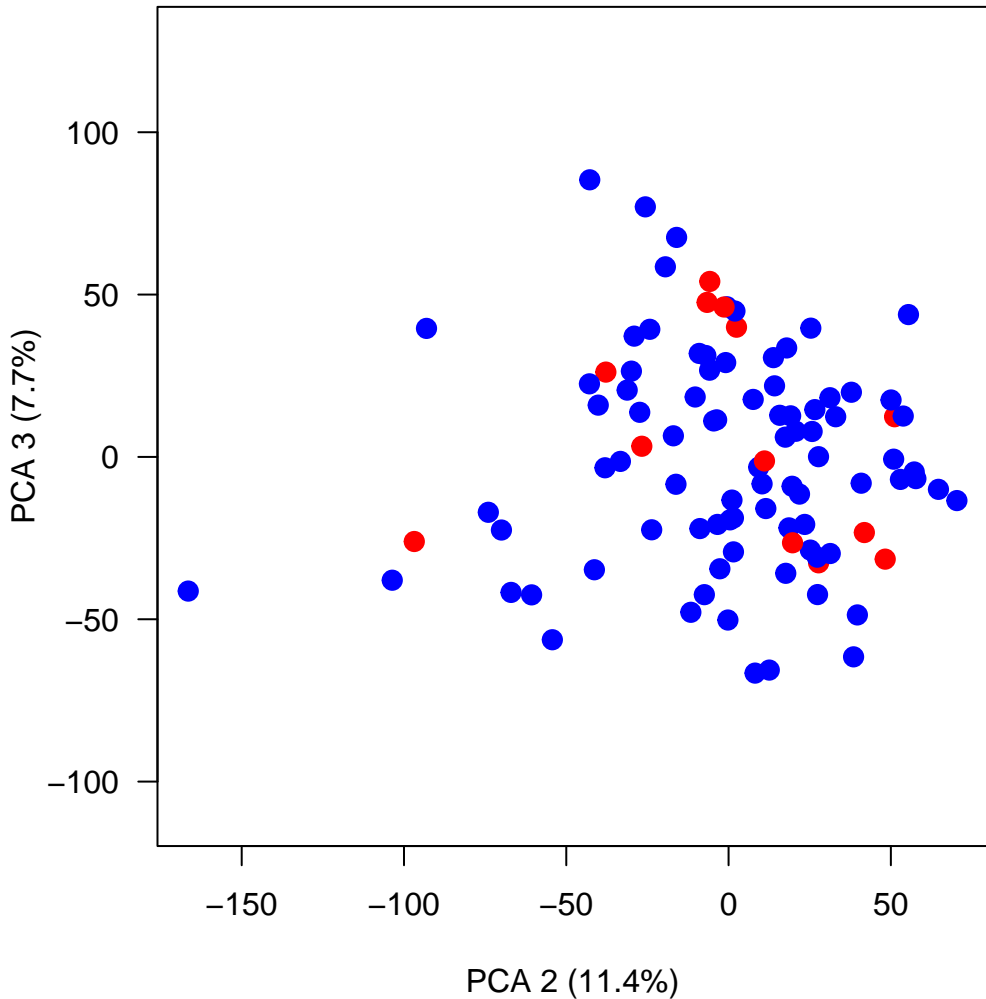

Supplement: Supplementary file 3 — Figure S2 [file EVA-17-e13755-s004.zip › Figure_S2B.pdf]

# PCA offspring

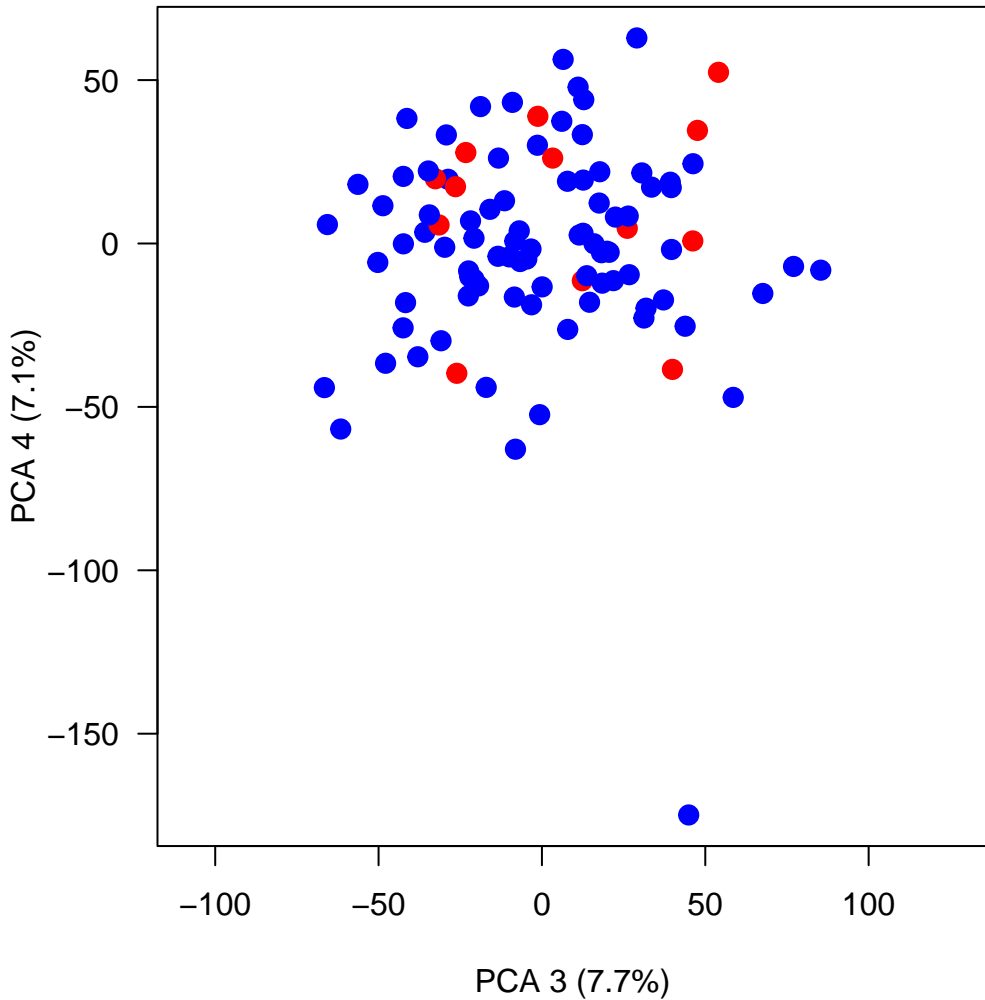

Supplement: Supplementary file 3 — Figure S2 [file EVA-17-e13755-s004.zip › Figure_S2C.pdf]

# PCA offspring

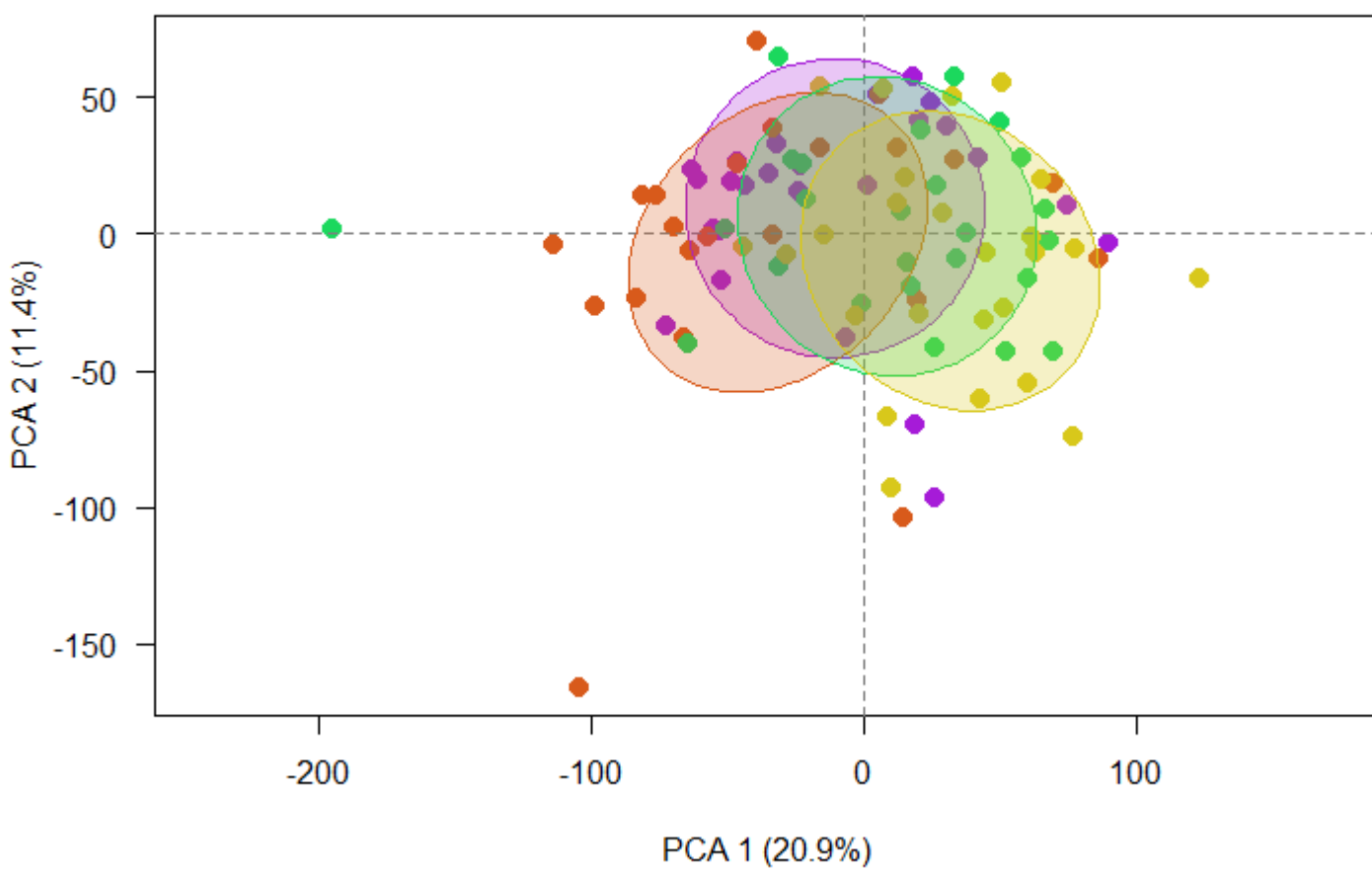

Supplement: Supplementary file 4 — Figure S3 [file EVA-17-e13755-s003.pdf]

# Pregnancy duration

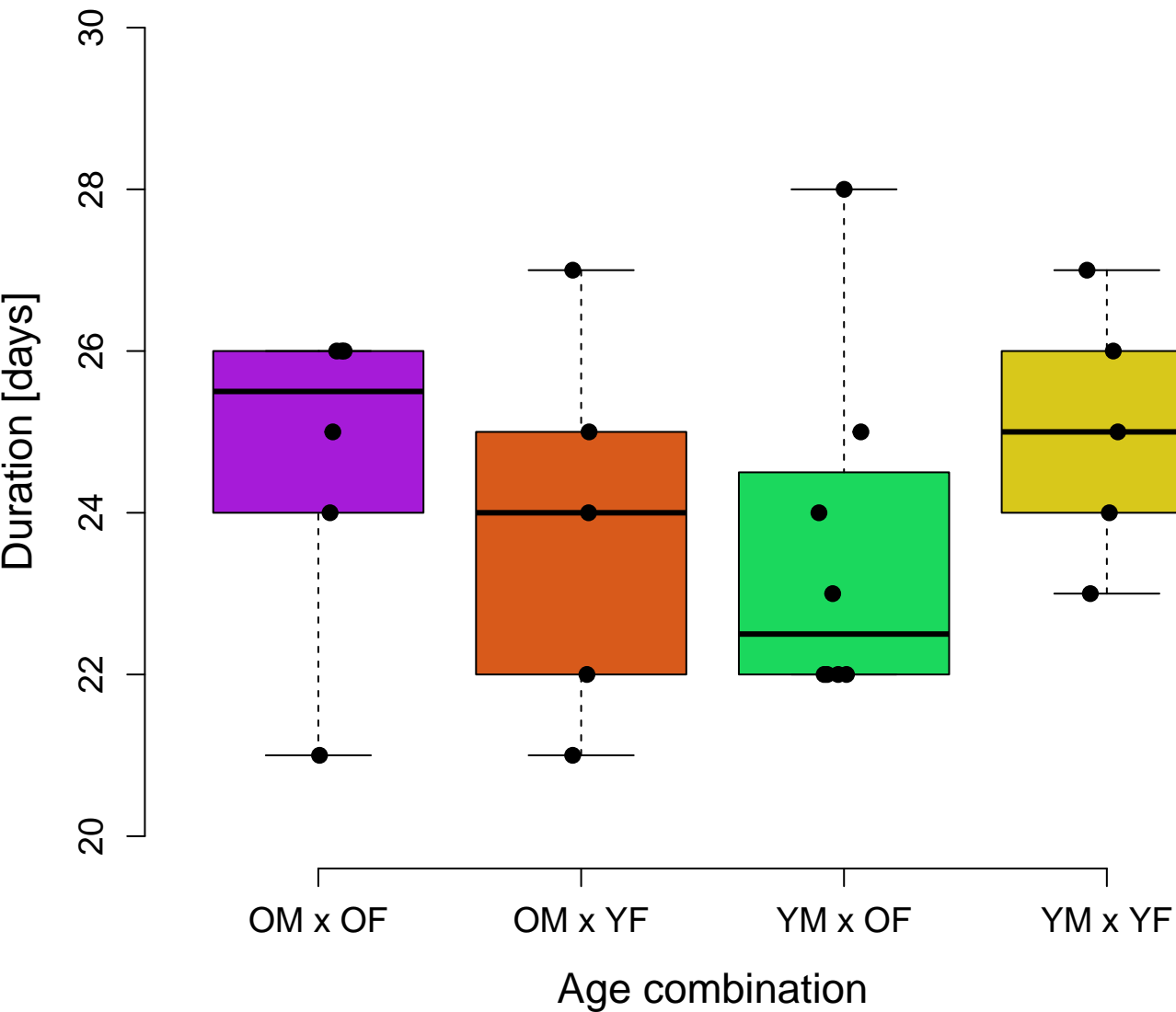

Supplement: Supplementary file 5 — Figure S4 [file EVA-17-e13755-s007.zip › Figure_S4A.pdf]

# Offspring count

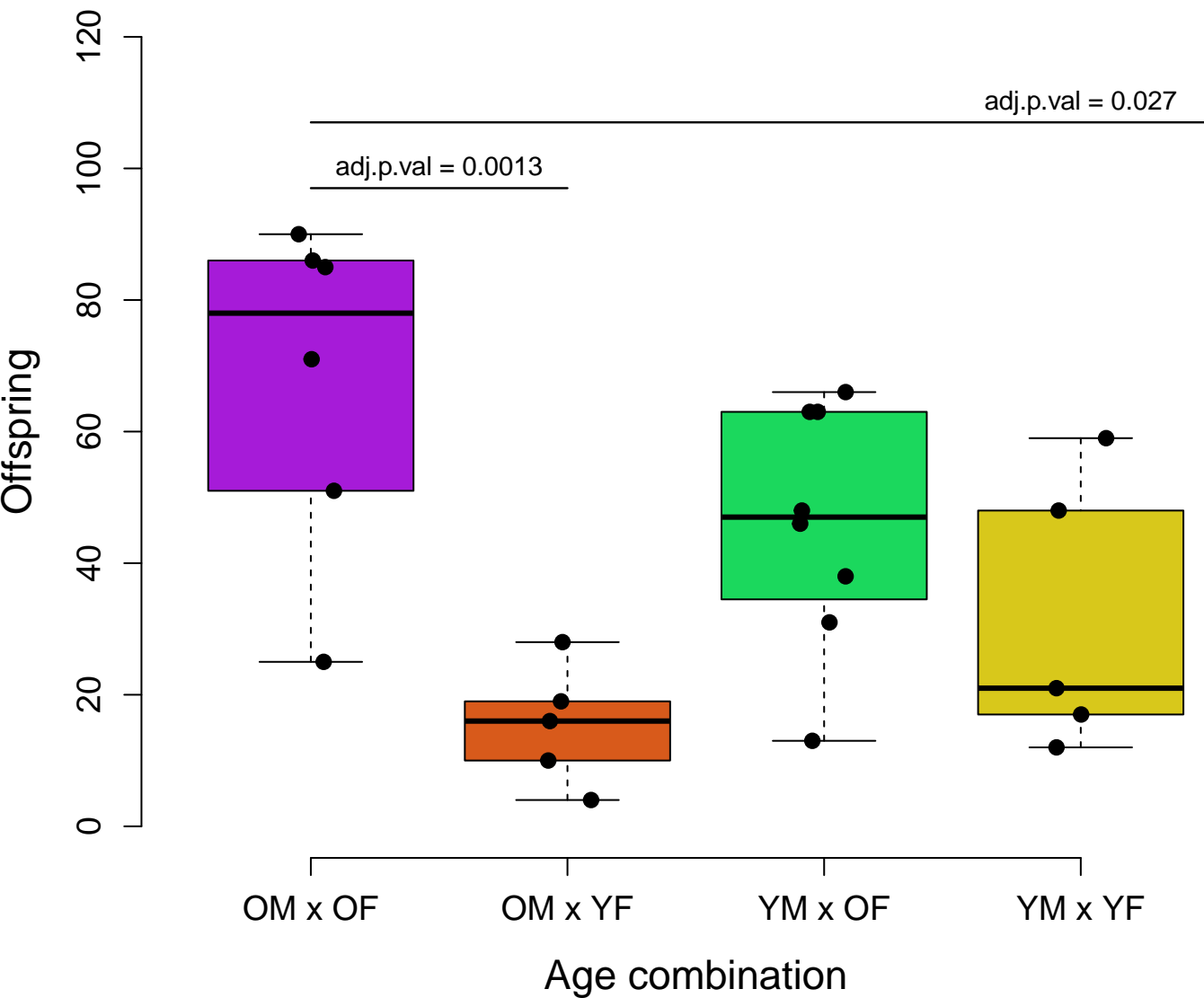

Supplement: Supplementary file 5 — Figure S4 [file EVA-17-e13755-s007.zip › Figure_S4B.pdf]

# Offspring length

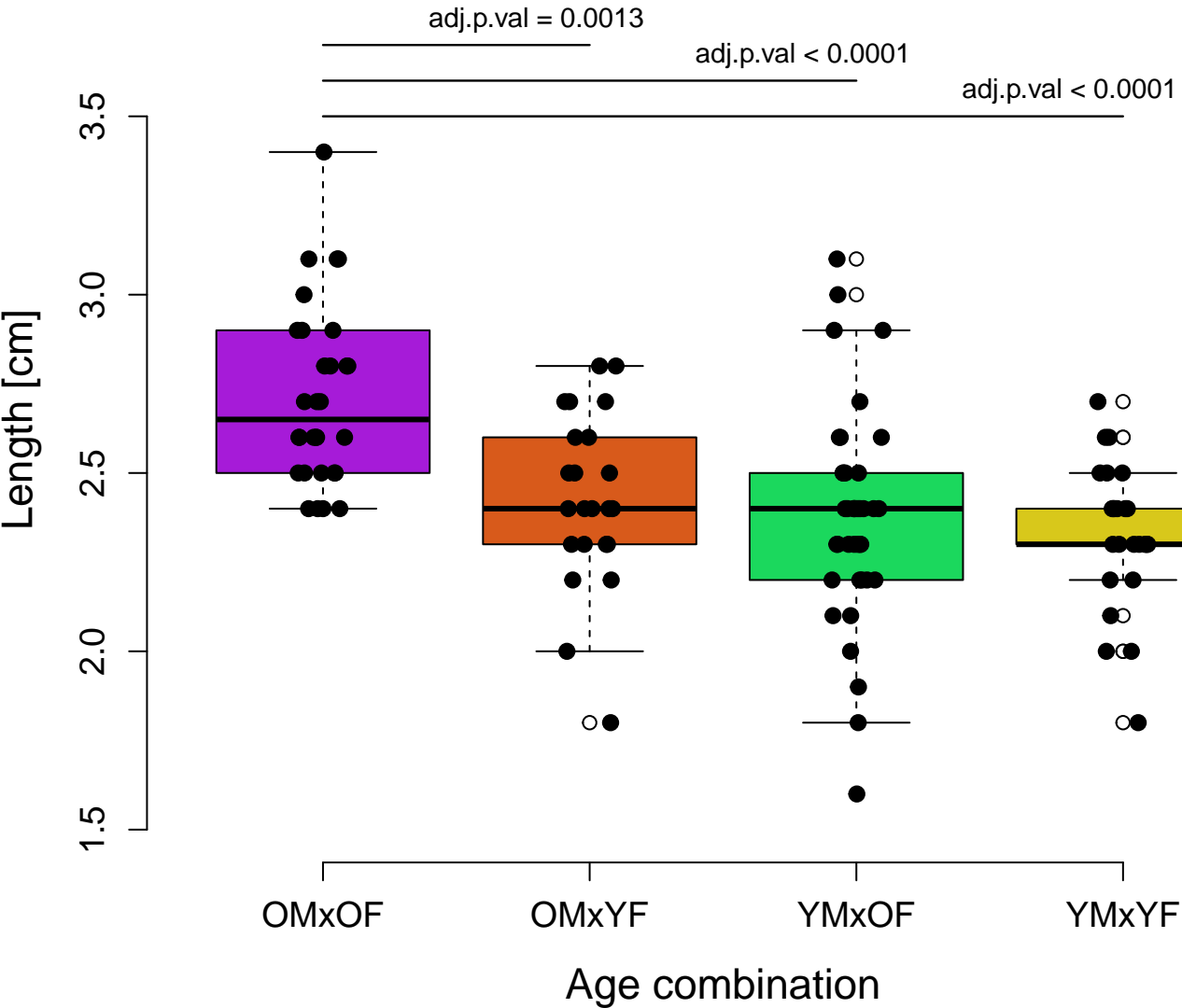

Supplement: Supplementary file 5 — Figure S4 [file EVA-17-e13755-s007.zip › Figure_S4C.pdf]
