# Supplementary material for: The effect of parental age on the quantity and quality of offspring in Syngnathus typhle, a species with male pregnancy
Source: Evol Appl. 2024 Jul 17;17(7):e13755. doi: 10.1111/eva.13755 (PMC11254578; doi:10.1111/eva.13755)
Supplement: Supplementary file 7 — Table S2 [file EVA-17-e13755-s006.zip › Table s2 caption.docx]

caption for Supporting Information Table S2:


Gene Set Enrichment Analysis (GSEA) results using g:Profiler conducted
in March 2023. The analysis inputted Danio rerio orthologue annotated
genes derived from Syngnathus typhle, based on differential gene
expression analysis of offspring RNAseq results across different
parental age combinations (see Table S1). The entries include a g:SCS
significance threshold of 0.05 and feature ENTREZGENE_ACC identifiers,
Gene Ontology biological process annotations, KEGG pathways, and
Reactome pathways.
